# Pt nanoclusters on GaN nanowires for solar-asssisted seawater hydrogen evolution

**DOI:** 10.1038/s41467-023-35782-z

**Published:** 2023-01-12

**Authors:** Wan Jae Dong, Yixin Xiao, Ke R. Yang, Zhengwei Ye, Peng Zhou, Ishtiaque Ahmed Navid, Victor S. Batista, Zetian Mi

**Affiliations:** 1 Department of Electrical Engineering and Computer Science, University of Michigan, 1301 Beal Avenue, Ann Arbor, MI 48109 USA; 2 Department of Chemistry and Energy Sciences Institute, Yale University, New Haven, CT 06520 USA

**Keywords:** Electrocatalysis, Energy, Chemical engineering, Photocatalysis

## Abstract

Seawater electrolysis provides a viable method to produce clean hydrogen fuel. To date, however, the realization of high performance photocathodes for seawater hydrogen evolution reaction has remained challenging. Here, we introduce n^+^-p Si photocathodes with dramatically improved activity and stability for hydrogen evolution reaction in seawater, modified by Pt nanoclusters anchored on GaN nanowires. We find that Pt-Ga sites at the Pt/GaN interface promote the dissociation of water molecules and spilling H* over to neighboring Pt atoms for efficient H_2_ production. Pt/GaN/Si photocathodes achieve a current density of −10 mA/cm^2^ at 0.15 and 0.39 V *vs*. RHE and high applied bias photon-to-current efficiency of 1.7% and 7.9% in seawater (pH = 8.2) and phosphate-buffered seawater (pH = 7.4), respectively. We further demonstrate a record-high photocurrent density of ~169 mA/cm^2^ under concentrated solar light (9 suns). Moreover, Pt/GaN/Si can continuously produce H_2_ even under dark conditions by simply switching the electrical contact. This work provides valuable guidelines to design an efficient, stable, and energy-saving electrode for H_2_ generation by seawater splitting.

## Abstract (web summary)

Seawater is the most abundant water source for hydrogen fuel production. Here the authors report a binary photoelectrode of Pt catalyst-GaN semiconductor with promising efficiency, productivity, and stability for seawater hydrogen evolution.

## Introduction

Hydrogen is a clean energy carrier that can replace fossil fuels due to its sufficiently high energy density and zero-carbon emission^1^. Among several possible methods, water electrolysis has been proposed as one of the most promising routes to produce high-purity H_2_ at low temperature^2^. A sustainable and green hydrogen economy could be realized by water electrolysis powered by renewable energy using seawater as an earth-abundant hydrogen source^3^. Acidic seawater, prepared by mixing an acid electrolyte with seawater, is an attractive aqueous solution for a highly efficient hydrogen evolution reaction (HER) because the concentration of protons near the cathode is very high. However, in acidic seawater, competition with both the chloride oxidation reaction (COR) and the oxygen evolution reaction (OER) is inevitable at the anode^4^. The COR is kinetically favored (although thermodynamically unfavored) when compared to the OER so the sluggish 4-electron kinetics of the OER leads to low O_2_ selectivity. The difference between the standard electrode potentials of the OER and COR can be gradually increased to 0.48 V by increasing the pH to up to 7.5^5^. So, the COR could be suppressed under alkaline conditions.

Electrocatalysts have been developed for H_2_ production by alkaline seawater splitting^6–11^. However, under alkaline conditions with pH > ~9.5, the high concentration of OH^−^ ions can lead to the precipitation of insoluble salts (e.g., Mg(OH)_2_ and Ca(OH)_2_)^5^. A viable approach to address the drawbacks of acidic and alkaline seawater is to employ neutral or weak-alkaline (7.5 < pH < 9.5) seawater. In neutral electrolytes, the kinetic bottleneck of the low HER activity is due to the Volmer step of water dissociation (H_2_O + e^−^ → H^*^ + OH^−^)^12^. In this regard, various electrocatalysts have addressed the H–OH cleavage in water molecules to enhance the HER under low overpotential^13–21^. However, the HER activity in neutral-pH seawater is still behind that in the acidic and alkaline electrolytes^22–27^.

Compared to the electrochemical method, photoelectrochemical (PEC) water splitting can achieve more efficient H_2_ evolution by integrating semiconductor and co-catalytic materials^28,29^. An electrical bias (e.g., from a solar cell) can positively shift the onset potential and promote hydrogen evolution with less electrical power consumption. In the past decades, many semiconductor materials have been developed and applied as photocathodes for solar water splitting^30–34^. However, conventional semiconductor materials such as Si, GaAs, and InP are easily corroded by surface holes accumulated under light illumination even in freshwater^35–37^. The stability of such semiconductors further deteriorates in seawater due to strong oxidants (e.g., chlorine and hypochlorite). Although protective films on photoelectrodes have improved the stability in freshwater^38,39^, to date, there have been a limited number of photocathodes that efficiently work in seawater^40,41^.

Earlier work has shown that N-terminated GaN nanowires (NWs) grown on Si effectively protect the underlying Si photocathode against degradation for >3000 h in acidic electrolytes^42,43^. Moreover, co-catalysts supported on GaN NWs have been shown to exhibit unique catalytic performance, likely originating from specific cocatalyst-GaN interactions^44–47^. Accordingly, the rational design of a cocatalyst/GaN binary system could lead to stable photocathodes for enhanced splitting of seawater.

In this work, we demonstrate the functionality of Pt nanoclusters (NCs) on GaN NWs that enhance the efficiency and stability of Si photocathodes as applied to seawater PEC HER. GaN NWs loaded with catalytic Pt NCs and grown on Si photocathodes catalyze water splitting while protecting the Si wafers from corrosion. Our DFT calculations show that Pt-Ga sites at the Pt/GaN interface promote the adsorption and activation of water molecules. The dissociated H^*^ atoms spill over to neighboring Pt NCs which facilitate H_2_ evolution. As a result, Pt/GaN/Si photocathode achieved a current density of −10 mA/cm^2^ at 0.15 and 0.39 V vs. RHE (V_RHE_) and high applied bias photon-to-current efficiency (ABPE) of 1.7% and 7.9% in seawater (pH = 8.2) and phosphate-buffered seawater (pH = 7.4), respectively. The photocathode exhibited a current density of −10 mA/cm^2^ at a small potential of −1.45 V vs IrO_*x*_ with a 2-electrode configuration, and operated steadily over a period of >120 h, indicating superior performance compared to photocathodes without GaN NWs. Under concentrated solar light (9 suns), a high current density of ~−169 mA/cm^2^ continuously generated high-purity H_2_.

## Results

### Microstructure and composition of Pt/GaN/Si

**Fig. 1:**
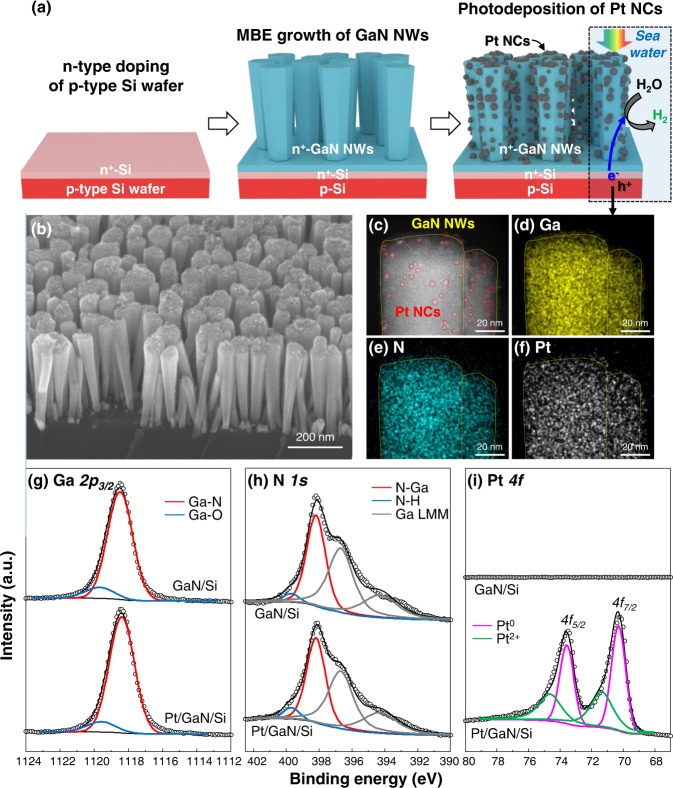
Pt nanoclusters on GaN nanowires grown on n^+^-p Si wafer. **a** Schematic illustration of the fabrication of Pt/GaN/Si photocathode by epitaxial growth of GaN nanowires (NWs) on Si p-n wafer and photodeposition of Pt nanoclusters (NCs). **b** 45°-tilted-view SEM image of Pt/GaN/Si. **c** HAADF-STEM image of Pt/GaN NWs. Bright contrast regions due to Pt NCs are marked with red circles. STEM-EDS elemental maps of **d** Ga, **e** N, and **f** Pt. **g** Ga *2p*_*3/2*_, **h** N *1* *s*, and **i** Pt *4f* XPS spectra for GaN/Si and Pt/GaN/Si electrodes.

GaN NWs were grown on an n^+^-p Si (100) substrate via plasma-assisted molecular-beam epitaxy (MBE) under nitrogen-rich conditions (Fig. 1a). Pt NCs coated the surface of GaN NWs, applied by photochemical deposition (More details of the experimental method are given in the method section). A scanning electron microscopy (SEM) image shows that GaN NWs were vertically aligned on the planar n^+^-p Si substrate with a length of ~400 nm and diameter of ~50 nm (Fig. 1b). Pt NCs are not clearly seen in the SEM image since they are too small.

We analyzed the microstructures of GaN NWs and Pt NCs by scanning transmission electron microscopy (STEM). In the high angular annular dark field (HAADF)-STEM image (Fig. 1c), Z-contrasts from the locally segregated Pt NCs were found on GaN NWs because of the large difference in atomic number between Pt (78) and Ga (31). The atomic distribution was elucidated by STEM-EDS elemental maps (Fig. 1d–f). We found that Ga, N, and Pt elements were uniformly distributed over the entire surface of GaN NWs. X-ray diffraction (XRD) patterns of Pt/GaN/Si showed GaN (002) and (004) peaks [JCPDS #02-1078] (Supplementary Fig. 1). However, the XRD peaks of Pt NCs couldn’t be detected because the crystallite Pt NCs are too small.

X-ray photoelectron spectroscopy (XPS) was performed to analyze the surface bonding states of GaN/Si and Pt/GaN/Si. Ga *2p*_*3/2*_ XPS spectra were deconvoluted with a major peak of Ga-N bond (1118.4 eV) and a minor peak of Ga–O or Ga–OH bond (1119.6 eV) (Fig. 1g)^48^. In the N 1s XPS spectra, the photoelectrons originating from N–Ga (398.2 eV) and N–H (399.7 V) were detected together with Ga LMM Auger electrons (Fig. 1h)^49–51^. The intensity and energy position of bonding states remained almost identical in the Ga *2p*_*3/2*_ and N *1s* spectra after the deposition of Pt NCs, indicating that N-rich GaN NWs were stable during the photodeposition against photochemical reaction in the aqueous solution. The Pt *4f* XPS spectrum, with two doublets of *4f*_*5/2*_ and *4f*_*7/2*_, was analyzed for Pt^0^ (71.3 and 74.5 eV) and Pt^2+^ (72.3 and 75.6 eV) bonds (Fig. 1i)^52^. Pt NCs consisted mainly of metallic Pt bonds with a small portion of Pt^2+^ bonds likely due to surface absorbed oxygen or Pt-N bond at the interface between Pt and GaN. For comparison, a control electrode of Pt/Si was prepared through photodeposition of Pt NCs on planar n^+^-p Si and has shown a Pt *4f* XPS spectrum with similar bonding states (Supplementary Fig. 2).

### Photoelectrochemical seawater splitting

**Fig. 2:**
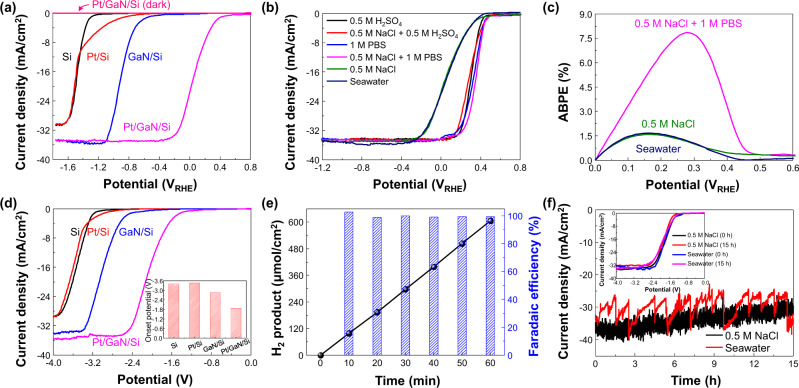
Photoelectrochemical seawater hydrogen evolution. **a** LSV curves of Si, Pt/Si, GaN/Si, and Pt/GaN/Si measured with 3-electrode configuration in 0.5 M NaCl solution under AM 1.5 G 1 sun illumination or in dark. **b** LSV curves of Pt/GaN/Si in six different aqueous solutions. Acidic solutions (pH = 0): 0.5 M H_2_SO_4_ and 0.5 M NaCl + 0.5 M H_2_SO_4_; Neutral solutions (pH = 7.4): 1 M PBS and 0.5 M NaCl + 1 M PBS; Weak alkaline solution: 0.5 M NaCl (pH = 9.1) and seawater (pH = 8.2). **c** ABPE of Pt/GaN/Si in seawater, 0.5 M NaCl, and 0.5 M NaCl + 1 M PBS. **d** LSV curves of Si, Pt/Si, GaN/Si, and Pt/GaN/Si measured with 2-electrode configuration in 0.5 M NaCl. Inset shows the onset potential of each electrode. **e** Amount of H_2_ produced and faradaic efficiency of Pt/GaN/Si in 0.5 M NaCl at −3 V vs IrO_x_. The faradaic efficiency is nearly 100%. **f** Stability of Pt/GaN/Si in 0.5 M NaCl and seawater at −3 V. The photocurrent density retains >85% of initial value after 15 h reaction. Inset shows the LSV curves before and after the stability test.

The PEC HER was performed by a simulated AM1.5 G solar spectrum incident on the Pt-decorated GaN NW array at an angle normal to the planar n^+^-p Si wafer (Supplementary Fig. 3a). Conduction band edge of GaN positions above the redox potential of HER, indicating appropriate energy band structure for electron transfer (Fig. S3b)^53–55^. An electrical bias was applied to the backside of the Si wafer. Linear sweep voltammetry (LSV) curves of Si, Pt/Si, GaN/Si, and Pt/GaN/Si were measured in 0.5 M NaCl (pH = 9.1) with a 3-electrode configuration under 1-sun (100 mW/cm^2^) illumination (Fig. 2a). The potentials at −10 mA/cm^2^ (η_10_) were −1.42, −1.41, −0.83, and 0.16 V_RHE_ for Si, Pt/Si, GaN/Si, and Pt/GaN/Si, respectively. Both GaN NWs and Pt NCs improved the activity of the PEC HER. The synergistic interaction between GaN NWs and Pt NCs played a vital role in achieving a high catalytic activity of Pt/GaN/Si. The saturated current densities of GaN/Si and Pt/GaN/Si (~35 mA/cm^2^) were larger than those without GaN NWs (~30 mA/cm^2^) because GaN NWs provide the enlarged surface area (Supplementary Figs. 4 and 5) and reduce the Fresnel reflection loss^56^. The Pt/GaN/Si showed negligible current density in dark due to the absence of the photogenerated charge carriers.

Pt/GaN/Si was evaluated in six different solutions (Fig. 2b) to investigate the effect of pH and the effect of the ions in the solution. Pt/GaN/Si exhibited high PEC HER activity (η_10_ > 0.3 V_RHE_) in 0.5 M H_2_SO_4_ solutions (pH = 0) and 1 M phosphate-buffered solutions (PBS) (pH = 7.4), even with dissolved NaCl. Despite having slightly worse HER performance than acidic and neutral solutions due to the lack of H^+^ ions near the electrode surface, the HER in 0.5 M NaCl (pH = 9.1) and seawater (pH = 8.2) could still be demonstrated at applied potentials > 0 V_RHE_. As a result, high ABPE of 7.9%, 1.6%, and 1.7% could be achieved in 0.5 M NaCl + 1 M PBS, 0.5 M NaCl, and seawater, respectively (Fig. 2c). On the other hand, the PEC HER performance of Pt/Si significantly degraded in 0.5 M NaCl and 0.5 M NaCl + 1 M PBS (Supplementary Figs. 6 and 7). These findings revealed that Pt/Si exhibited poor catalytic activity due to the sluggish kinetics of water dissociation in the neutral electrolyte. Moreover, the LSV curves of Pt/GaN/Si were identical in solutions with different NaCl concentrations, confirming feasibility for seawater hydrogen evolution (Supplementary Figs. 8 and 9).

In order to test for practical photoelectrolysis, LSV curves were measured in 0.5 M NaCl solution with a 2-electrode configuration *vs*. iridium oxide (IrO_x_) counter electrode (Fig. 2d). η_10_ was −3.41, −3.47, −2.85, and −1.88 V for Si, Pt/Si, GaN/Si, and Pt/GaN/Si, respectively. η_10_ could be improved to −1.33 and −1.45 V when measured in acidic (0.5 M NaCl + 0.5 M H_2_SO_4_) and phosphate-buffered (0.5 M NaCl + 1 M PBS) seawater, respectively (Supplementary Fig. 7). To the best of our knowledge, −1.45 V is the best η_10_ among the PEC and EC HER electrodes in neutral-pH seawater (Supplementary Table 1). We further evaluated the faradaic efficiency of H_2_ and O_2_ produced from Pt/GaN/Si with a 2-electrode configuration in 0.5 M NaCl and seawater. At −2 V *vs* IrO_x_, H_2_ production rate was 275 μmol/cm^2^/h and O_2_ production rate was 128 μmol/cm^2^/h in 0.5 M NaCl (Supplementary Fig. 10a). The H_2_/O_2_ ratio (2.15) nearly approaches to the theoretical value (2) for water splitting. When a larger potential (−3 V vs IrO_*x*_) was applied to the photocathode, H_2_ and O_2_ production rates were 600 and 216 μmol/cm^2^/h in 0.5 M NaCl (Fig. 2e and Supplementary Fig. 10b), and 575 and 159 μmol/cm^2^/h in seawater (Supplementary Fig. 10c), respectively. Regardless of electrolyte and applied potential, faradaic efficiency of H_2_ was almost 100%, confirming the high efficiency of electron utilization. Meanwhile, faradaic efficiency of O_2_ was decreased from 93% to 61–75% by increasing the potential from −2 to −3 V vs IrO_*x*_ likely due to Cl^−^ oxidation reactions. However, it should be noted that the O_2_ selectivity at the anode could be hardly controlled by designing the photocathode.

Pt/GaN/Si revealed excellent stability with little degradation in chronoamperometric (CA) and LSV curves after reaction for 15 h in 0.5 M NaCl and seawater (Fig. 2f, Supplementary Figs. 11 and 12). There was no noticeable change in morphology and microstructure of Pt/GaN/Si (Supplementary Fig. 13). Compared with the rapid degradation of Pt/Si, clearly, the physical and chemical stability of Si photoelectrodes with GaN NWs and Pt NCs is greatly improved (Supplementary Fig. 14). Furthermore, the performance of Pt/GaN/Si could be fully recovered after the re-deposition of Pt NCs, indicating that removal of Pt NCs in seawater was the reason for the degradation (Supplementary Fig. 15)^57^. Although the catalytic activity was slightly decreased with a thin TiO_2_ layer (2 nm) on Pt/GaN/Si (Supplementary Fig. 16), Pt NCs could be stabilized on GaN NWs, leading to long-term stability for >120 h in pH neutral seawater.

### Theoretical modeling

**Fig. 3:**
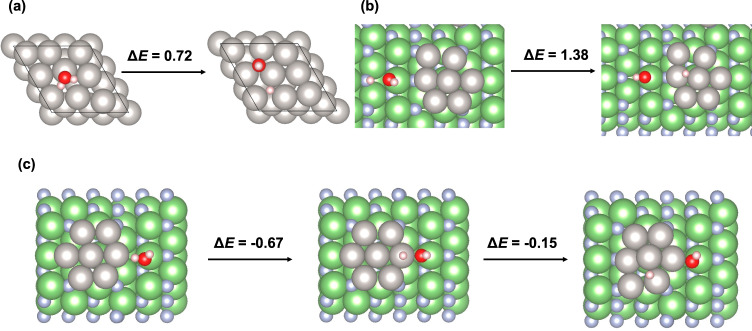
Theoretical study of water dissociation. The Pt/GaN interface promotes water splitting. Optimized structures and calculated energy changes of **a** water dissociation on Pt(111), **b** proton transfer from surface N–H to a Pt cluster, **c** water dissociation at a Pt-Ga site at the Pt/GaN interface and subsequent H spillover to Pt surface. All energy changes are in the unit of eV. The white, red, blue, green, and gray spheres represent H, O, N, Ga, and Pt atoms, respectively.

We explored the mechanistic role played by the Pt/GaN binary system via periodic density function theory (DFT) calculations with the Vienna Ab Initio Simulation Package (VASP)^58–61^ (Details of the computational modeling are given in the method section and Supplementary Fig. 17). The proton reduction by Pt and other catalysts is well known in literature^62,63^. However, Pt is not good at water reduction in neutral or alkaline solution since the water dissociation step is the bottleneck for efficient hydrogen evolution under those conditions^12,20^. Therefore, we focused on the water dissociation step on Pt(111) and Pt/GaN interface to provide mechanistic understanding of the good performance of our Pt/GaN binary system. We first analyzed the dissociation of water on the Pt(111) surface. The H–OH bond cleavage is highly endothermic on Pt(111), with an energy change of 0.72 eV (Fig. 3a). This is consistent with the experimental fact that Pt is not an effective catalyst for H_2_ production in neutral or alkaline solutions. Water is known to dissociate on the GaN m-plane due to the high polarity of the surface. When a water molecule approaches the GaN$(10\bar{1}0)$ surface, it spontaneously dissociates to H^+^ and OH^−^. The OH^−^ coordinates to Ga and H^+^ binds to N. However, the transfer of a proton from the surface N–H group to the Pt cluster was found to be highly endothermic by 1.38 eV (Fig. 3b), probably due to the high pKa of NH groups. At the same time, we found that water dissociation at a Pt-Ga site of the Pt/GaN interface is quite favorable, with an energy change of −0.67 eV (Fig. 3c). We note that the gallium oxynitride (GaON) layer can be formed on the surface of GaN under the condition of photoelectrochemical water splitting^43^. In order to investigate the dependence of water dissociation energetics on GaN and GaON surface, we studied water dissociation at Pt-Ga site of the Pt/GaON interface. The energy change of water dissociation is similar to that at the Pt/GaN interface (Supplementary Fig. 18). Compared to water dissociation on Pt(111), the dissociation of water at a Pt-Ga site benefits from the asymmetric atomic environment that facilitates the heterolytic cleavage of the H–OH bond to form Pt–H and Ga–OH. The *H spontaneously spills over to neighboring Pt atoms, with a Δ*E* of −0.15 eV. So, the overall energy change due to water splitting at the Pt/GaN interface is −0.81 eV. Effective water dissociation at the Pt-Ga site is likely responsible for the high activity of seawater splitting on Pt/GaN/Si in neutral pH or weakly alkaline solutions. Interestingly, the difference in water dissociation energy Δ*E* between Pt(111) and Pt-Ga site is 1.53 eV, similar to the difference in overpotential (Δη_10_ = 1.58 V) between the Pt/Si and Pt/GaN/Si electrodes, suggesting the importance of Pt-Ga sites at the Pt/GaN interface for achieving efficient water splitting.

### Concentrated solar light PEC seawater splitting

**Fig. 4:**
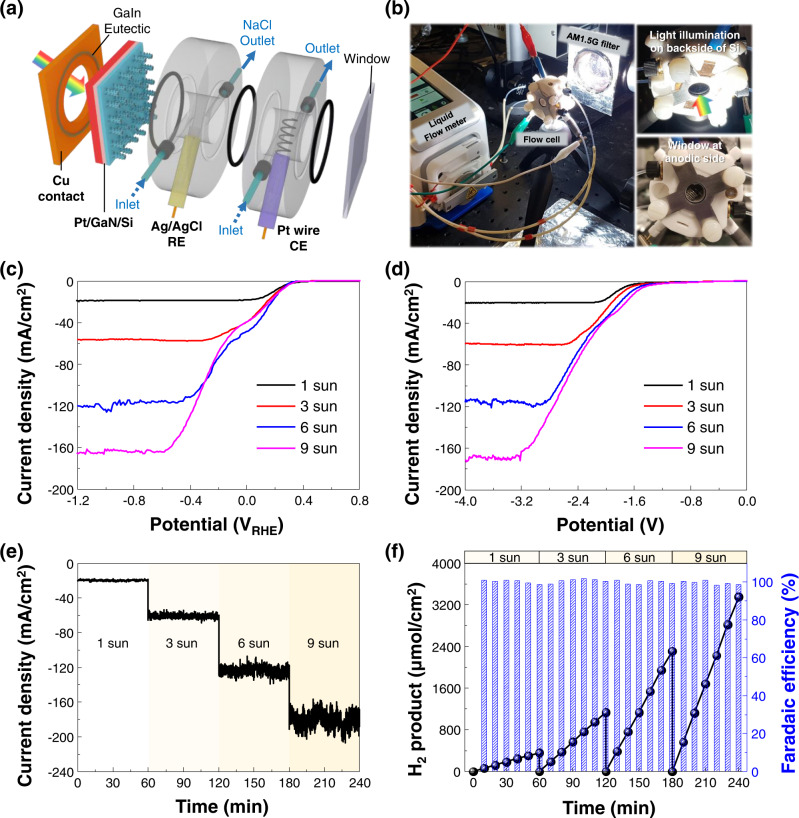
Concentrated solar light PEC water splitting. **a** Schematic illustration and **b** photographs of the liquid flow cell for PEC water splitting. Light illuminates on the backside of n^+^-p Si wafer and HER takes place on the front side of Pt/GaN nanowires (NWs). LSV curves of Pt/GaN/Si measured with **c** 3-electrode and **d** 2-electrode configurations in 0.5 M NaCl under different light intensities. **e** Chronoamperometric curve and **f** amount of H_2_ produced and faradaic efficiency measured under light intensities of 1, 3, 6, and 9 suns at −3 V.

Concentrated solar light can enhance the number of photogenerated electrons in the semiconductor and increase the photocurrent for PEC HER. However, the reaction kinetics under concentrated solar light with vigorous H_2_ evolution may be limited by mass transport and shielding of active sites by gaseous bubbles. A flow cell can simultaneously solve these two issues by fast delivery of the reagent and the physical detachment of gas bubbles from the electrode surface. Hence, we prepared a liquid flow cell reactor for the concentrated solar light experiment to demonstrate high photocurrent density (Fig. 4a). Light was incident on the backside of n^+^-p Si wafer while the PEC HER occurred on the front side in the reactor (Fig. 4b and Supplementary Movie 1). 0.5 M NaCl solution continuously flowed at a rate of 20 ml/min during the measurement. Pt/GaN/Si revealed η_10_ of ~0.2 V_RHE_ and saturated current density of ~−19 mA/cm^2^ under 1 sun light (Fig. 4c). As the light was intensified to 3, 6, and 9 suns, the saturated current density increased to ~−58, ~−118, and ~−163 mA/cm^2^, respectively. In the 2-electrode configuration, Pt/GaN/Si also showed a gradual increase in the photocurrent density, reaching a high value of ~−165 mA/cm^2^ at −3.2 V under 9 suns light illumination (Fig. 4d). The linear correlation between the light intensity and the saturation current density indicates that the light intensity and the number of photogenerated electrons in the photoelectrode are the limiting factors for PEC HER in the flow cell.

CA measurements were evaluated for 4 h under 1, 3, 6, and 9 suns at −3 V vs IrO_*x*_ (Fig. 4e). The current densities for each period of reactions (−20, −60, −121, and −169 mA/cm^2^, respectively) were stable in 0.5 M NaCl. This is the record high photocurrent density for PEC HER among photocathodes in aqueous electrolytes (Supplementary Table 2). The amount of H_2_ product and faradaic efficiency were monitored for 4 h at −3 V under different light intensities (Fig. 4f). The production rate gradually increased from 359 to 3351 μmol/cm^2^/h as the incident illumination was intensified from 1 to 9 suns. The faradaic efficiency was nearly 100% regardless of the light intensity, confirming that all photocurrents participated in HER. Moreover, two individual photocathodes were tested for 4 h at −3 V under 9 suns in 0.5 M NaCl (Supplementary Fig. 19). Both samples showed average photocurrent densities of 166 and 157 mA/cm^2^ without degradation, indicating remarkable stability even with vigorous gas bubbling. Furthermore, the concentrated solar light experiment was conducted in phosphate-buffered solution (0.5 M NaCl + 1 M PBS) and exhibited high photocurrent density (>160 mA/cm^2^), near-unity faradaic efficiency (~100%), and positive shift of potential by ~0.1 V (Supplementary Fig. 20). Therefore, Pt/GaN/Si has been proven to be a highly efficient, selective, and stable photocathode for seawater splitting.

### PEC (light)/EC (dark) switchable electrode

**Fig. 5:**
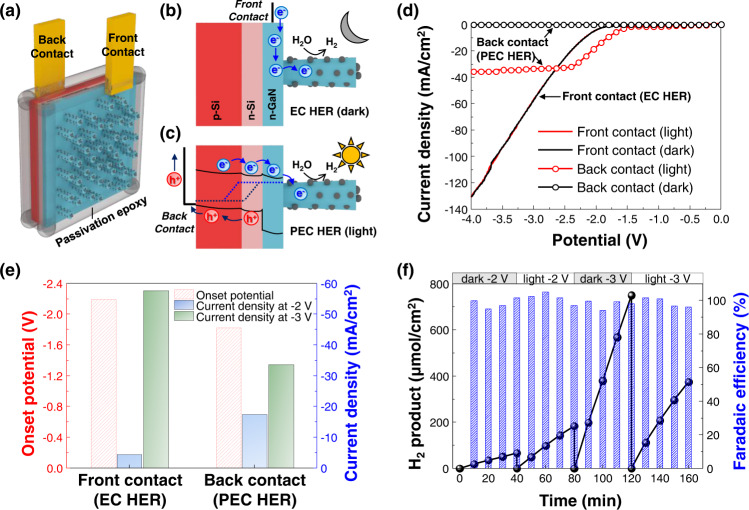
PEC/EC switchable electrode. **a** Schematic of switchable dual contact electrode. The front contact is placed on the front side of GaN nanowires (NWs) and the back contact is placed on the back side of n^+^-p Si wafer. GaIn eutectic was sandwiched between the Cu contacts and the substrate for Ohmic connection. Illustrations of working principles of **b** front contact for electrochemical HER under dark and **c** back contact for photoelectrochemical HER under light. **d** LSV curves of electrode measured with 2-electrode configuration in 0.5 M NaCl solution. **e** Onset potential and current density at −2 and −3 V for front and back contacts. Onset potential was defined as the potential at −10 mA/cm^2^. **f** Amount of H_2_ produced and faradaic efficiency measured at −2 and 3 V under dark (front contact) and light (back contact) conditions.

Our GaN NWs were degenerately doped n-type and highly electrically conductive with the loading of large densities of Pt NCs^53^. Hence, the electrical contact at the front side of GaN NWs/Si wafer established a cathode for electrochemical HER (Supplementary Figs. 21 and 22). Therefore, we found that Pt/GaN/Si could work under either light or dark conditions by simply switching the contact positions (Fig. 5a). During the EC HER in the dark, the electrical bias injects electrons into GaN NWs, which drift to Pt NCs leading to H_2_ evolution (Fig. 5b). In contrast, during the PEC HER with light, the n^+^-p Si substrate with a narrow bandgap (∼1.1 eV) generates electron-hole pairs by solar irradiation (Fig. 5c). The photogenerated electrons in the conduction band migrate toward *n*-type GaN NWs and Pt NCs for the HER whereas holes move to the back contact and counter electrode and engage in the anodic reaction. We assessed the performance of our switchable electrode in 0.5 M NaCl solution (Fig. 5d). Importantly, there was no photoresponse when the front contact was in use, i.e., when operating in the EC mode; LSV curves under dark and light conditions overlapped (Supplementary Fig. 23). On the contrary, when the back contact was used (i.e., PEC mode), there was no photocurrent in the dark, whereas η_10_ was 0.11 V_RHE_ in a 3-electrode configuration and −1.83 V in a 2-electrode configuration under 1 sun light irradiation, respectively. The built-in-potential of the Si p-n junction can shift positively the onset potential by ~0.4 V compared to the EC reaction and realize a relatively high current density (−17.5 mA/cm^2^) at a low operating voltage (−2 V) (Fig. 5e). Yet, the photocurrent density was saturated to ~35 mA/cm^2^ limited by minority carrier diffusion in reverse bias as well as the number of photogenerated charge carriers. As the EC HER reaction was performed using the front contact in the dark, there was a higher yield of H_2_ (−58 mA/cm^2^) at −3 V. The high quality of degenerately doped n-type GaN NWs, evidenced by the enhancement of saturation photocurrent density of the Pt/GaN/Si assembly in comparison with Pt/Si, allows for the usage of majority carriers for HER due to factors such as the absence of Fermi level pinning and efficient electron transport to Pt at the non-polar sidewalls^43^. Furthermore, we have measured the productivity and faradaic efficiency of Pt/GaN/Si at −2 and −3 V under dark and light conditions (Fig. 5f and Supplementary Fig. 24). At −2 V, the production rate was 100 and 276 μmol/cm^2^/h under dark and light, respectively, showing that the PEC HER is more favored than the EC HER under light illumination. On the other hand, at the more negative potential of −3 V, the production rate of EC HER (1125 μmol/cm^2^/h) was ~2 times higher than the PEC HER (562 μmol/cm^2^/h). Remarkably, the faradaic efficiency was almost 100% for all conditions. Therefore, the Pt/GaN/Si assembly can work as a photocathode during the day and as an electrocatalyst at night for continuous generation of clean hydrogen.

## Discussion

In summary, we have demonstrated that Pt NCs bound to GaN NWs are highly active for the PEC HER in acidic, neutral, and weak alkaline solutions of seawater. Our DFT calculations further confirm that the Pt-Ga sites at the Pt/GaN interface promote the dissociation of water and facilitate H_2_ evolution. The Pt/GaN on Si electrodes is thus a synergetic binary system for achieving highly efficient catalytic hydrogen evolution over a wide range of pH. Pt/GaN/Si exhibited superior η_10_ = 0.15, 0.16 and 0.39 V_RHE_ with a high ABPE = 1.7%, 1.6% and 7.9% in seawater (pH = 8.2), 0.5 M NaCl (pH = 9.1), and phosphate-buffered seawater (pH = 7.4), respectively. In the 2-electrode configuration, the photocathode operated over 120 h and generated a record-high photocurrent density of ~−169 mA/cm^2^ under concentrated solar light (9 suns). The measured efficiency and stability are among the highest values ever reported for the PEC HER in seawater. Importantly, we find that Pt/GaN/Si can uniquely work in both light (PEC) and dark (EC) conditions. The reported findings show that the Pt/GaN/Si assembly promotes catalytic reactions and can be used as an energy-saving electrode, with superior performance when compared to conventional photoelectrolytic systems.

## Methods

### Fabrication of GaN/Si photocathode

n^+^-p Si junction was fabricated through a standard thermal diffusion process using (100) Si wafer. Phosphorus was spin-coated as an n-type dopant on front side of the double-side polished p-type Si (100) wafer, and boron was spin-coated on the other side as a p-type dopant for Ohmic metal contact. The spin-coated wafer was then annealed at 950 °C under nitrogen atmosphere for 4 h. Plasma-assisted molecular beam epitaxy was used for the growth of GaN NWs on the n^+^ side of the n^+^-p Si wafer under nitrogen rich-condition with a N_2_ flow rate of 1.0 standard cubic centimeter per minute. The substrate temperature was held at 790 °C and the growth duration was ~2 h. The forward plasma power was 350 W with Ga flux beam equivalent pressure (BEP) of 5 × 10^−8 ^Torr.

### Photochemical deposition of Pt nanoclusters

The as-grown GaN/Si was placed on a Teflon holder in the glass chamber with a quartz window. 10 μl of 0.2 M chloroplatinic acid hydrate (99.9%, Sigma Aldrich), 55 ml of deionized (DI) water, and 11 ml methanol were poured into the glass chamber. The chamber was evacuated using a vacuum pump for 5 min. Then, the sample was irradiated using a 300 W xenon lamp for 30 min. Platinum ions from chloroplatinic acid hydrate were reduced and deposited onto GaN NWs during the light illumination.

### TiO_2_ passivation layer

2 nm TiO_2_ passivation layer was deposited on the Pt/GaN/Si via thermal atomic layer deposition (ALD) at 250 °C with tetrakis(dimethylamino) titanium as the precursor.

### Preparation of contacts

Small droplets of GaIn eutectic alloy were placed on the front and back surfaces of GaN/Si. Then, Cu wires were connected on both sides. The backside, the electrical connection on the front side, and the edges of the electrodes were passivated by epoxy (Loctite EA-615). The sample areas were between 0.05 and 0.1 cm^2^.

### Characterization

SEM analysis was conducted using MIRA3 TESCAN with an accelerating voltage of 10 kV. STEM and STEM-EDS images were collected at 200 kV using JEOL 2100 F microscope with Cs-corrector. XPS was measured using a Kratos Axis Ultra XPS with a monochromatic Al Kα source. XRD measurements were done using Rigaku 300.

### Photo/electrochemical measurements

A reactor made of quartz was used for the measurement. Working electrode, counter electrode, and/or reference electrode were placed in the reactor filled with electrolyetes. LSV measurements were conducted with 2-electrode or 3-electrode configurations using a potentiostat. For the 2-electrode measurements, iridium oxide (IrO_*x*_) was used as the counter electrode. For the 3-electrode measurements, Ag/AgCl filled with 3 M KCl was used as the reference electrode. The electrolytes used for PEC measurements were 0.5 M H_2_SO_4_, 0.5 M NaCl + 0.5 M H_2_SO_4_, 1 M phosphate-buffered solution (PBS), 0.5 M NaCl + 1 M PBS, NaCl solutions with different concentrations, and artificial seawater. 0.5 M H_2_SO_4_ was prepared by diluting H_2_SO_4_ (Sigma Aldrich) with DI water. 1 M PBS was used as purchased (Sigma Aldrich). NaCl (Sigma Aldrich) was dissolved in the solutions to concentrations of 0.1–5.0 M. The artificial seawater was prepared by dissolving the sea salts (manufactured by Instant Ocean) in fresh water. The measured potential (V_Ag/AgCl_) (V) can be converted to the reversible hydrogen electrode (V_RHE_) (V) by using the Nernst function: V_RHE_ = V_Ag/AgCl_ + 0.197 + 0.0592 × pH. pH values of electrolytes were measured using a pH meter (Metttler Toledeo). The light source used for the illumination was LCS-100 (ORIEL) and the light intensity with AM 1.5 G filter was calibrated by adjusting the distance from sample to light source. The back contact was used for the PEC measurements and the front contact was used for the EC measurements. All measurements were conducted under ambient pressure at room temperature. For the gas analysis, the reactor was separated from outside air by tightly closing the lid, and then the reaction was carried out. After the reaction, 1 ml of gas in the reactor was injected to gas chromatographer (Shimadzu GC-8A) equipped with thermal conductivity detector.

### Concentrated solar light photoelectrochemical measurements

The intense simulated solar light (1–9 suns) was illuminated on the backside of the photoelectrodes through a hole at the center of Cu back contact and reaction was conducted on the front side (Pt/GaN NWs side) in a flow cell with Pt wire counter electrode and Ag/AgCl reference electrode. Ga-In eutectic was sandwiched between Cu back contact and n^+^-p Si wafer for ohmic contact. Liquid flow rate of 0.5 M NaCl solution was 20 ml/min circulated by a Masterflex Ismatec microflow pump (Cole-Parmer). Liquid in the cathodic reactor continuously circulated in the closed system connected to a sealed chamber. The collected H_2_ product in the sealed chamber was analyzed.

### Computational section

#### General setup

We performed all the periodic boundary condition DFT calculations using the Vienna Ab initio Simulation Package (VASP)^58–61^ version 5.4. The Perdew-Burke-Ernzerhof (PBE)^64^ exchange-correlation functional and the projected-augmented wave (PAW) method^65,66^ were used to describe the ion-electron interactions. The spin-polarized Kohn-Sham calculations were performed for all calculations. Dispersion interactions were considered using Grimme’s empirical dispersion correction version 3 with the Becke-Johnson damping (D3-BJ). The cutoff energy for the plane wave basis function was set to 450 eV. The Gaussian smearing method and a smearing parameter of *σ* = 0.1 eV were used to facilitate the SCF convergence. The SCF convergence criterion was set to be 10^−6^ eV per unit cell.

#### Bulk structure optimization

Both the unit cell size and shape, as well as the ionic positions were allowed to relax in the optimization of bulk structures of GaN and Pt. The geometry convergence criterion was set to be an energy change <10^−5^ eV per unit cell between two consecutive calculations. A 9 × 9 × 9 Monckhorst-Pack (MP) type k-point grid^67^ was used for the geometry optimization of GaN and Pt bulk structures.

#### Surface reaction calculations

We constructed slab models for the GaN$(10\bar{1}0)$ and Pt(111) facets. A vacuum layer of 20 Å was used to eliminate the interaction between periodic images. A supercell of 8.33 Å × 8.33 Å × 29.07 Å was used for the Pt(111) slab with four layers of Pt, and a supercell of 15.63 Å × 9.59 Å ×  35.00 Å was used for the GaN$(10\bar{1}0)$ slab with 6 GaN layers. A Pt_7_ cluster taken from the Pt(111) surface was used to model small Pt clusters. The Pt/GaN interface was modeled with the Pt_7_ cluster on top of the GaN$(10\bar{1}0)$ surface (Supplementary Fig. 17). We also constructed Pt7@GaON surface by replacing the surface N atoms with O atoms to study water dissociation energetics at the Pt and GaON interfaces. A 3 × 3 × 1 Monckhorst-Pack (MP) type k-point grid^67^ was used in all slab calculations of Pt(111), Pt7@GaN$(10\bar{1}0)$, and Pt7@GaON$(10\bar{1}0)$. During the geometry optimization, the top two layers of atoms, and the adsorbed molecules were allowed to relax while the bottom several layers of atoms were fixed at their bulk position. The convergence criterion for geometries optimization was set to be 0.02 eV/Å.

## Supplementary information

Supplementary Information

Description to Additional Supplementary Information

Supplementary Video 1

## Data Availability

All other data supporting the findings in this study, as well as the Supplementary Information files, are available from the corresponding authors upon reasonable request.
