# Supplementary material for: Pt nanoclusters on GaN nanowires for solar-asssisted seawater hydrogen evolution
Source: Nat Commun. 2023 Jan 12;14:179. doi: 10.1038/s41467-023-35782-z (PMC9837051; doi:10.1038/s41467-023-35782-z)
Supplement: Supplementary file 1 — Supplementary Information [file 41467_2023_35782_MOESM1_ESM.pdf]

Supplementary Information

Pt Nanoclusters on GaN Nanowires for Solar-Assisted Seawater Hydrogen Evolution

Wan Jae Dong,[1,‡] Yixin Xiao,[1,‡] Ke R. Yang,[2,‡] Zhengwei Ye,[1] Peng Zhou,[1] Ishtiaque Ahmed Navid,[1] Victor S. Batista,[2,*] Zetian Mi[1,*]

[1] Department of Electrical Engineering and Computer Science, University of Michigan, 1301 Beal Avenue, Ann Arbor, Michigan 48109, USA

[2] Department of Chemistry and Energy Sciences Institute, Yale University, New Haven, Connecticut 06520, USA

‡W. J. Dong, Y. Xiao, K. R. Yang contributed equally to this work.

*E-mail: ztmi@umich.edu, victor.batista@yale.edu

X-ray diffraction patterns of Si, GaN/Si, and Pt/GaN/Si

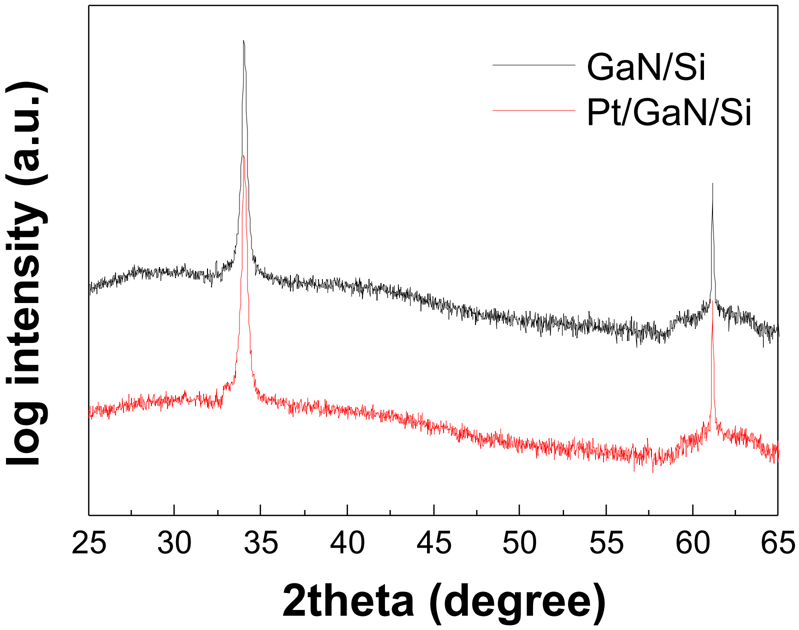

Supplementary Fig. 1. X-ray diffraction (XRD) patterns of GaN/Si and Pt/GaN/Si. XRD peaks of GaN (002) and (004) were observed.

X-ray photoelectron spectroscopy of Si and Pt/Si

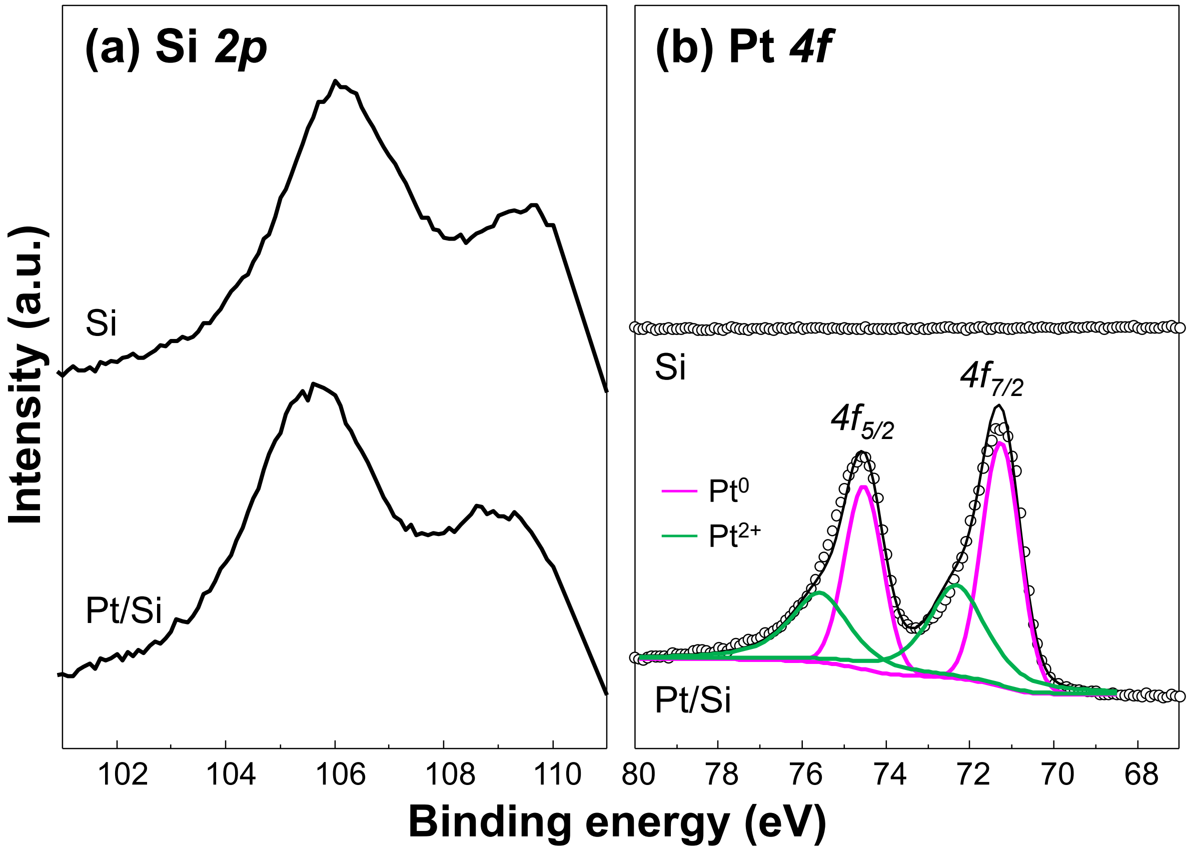

Supplementary Fig. 2. X-ray photoelectron spectroscopy (XPS) spectra of (a) Si *2p* and (b) Pt *4f* for Si and Pt/Si. Si *2p* peaks were found in both samples and Pt *4f* peaks were found only in Pt/Si, indicating that Pt cocatalyst was deposited on n^+-p Si wafer by photodeposition.

Reactor and energy band diagram

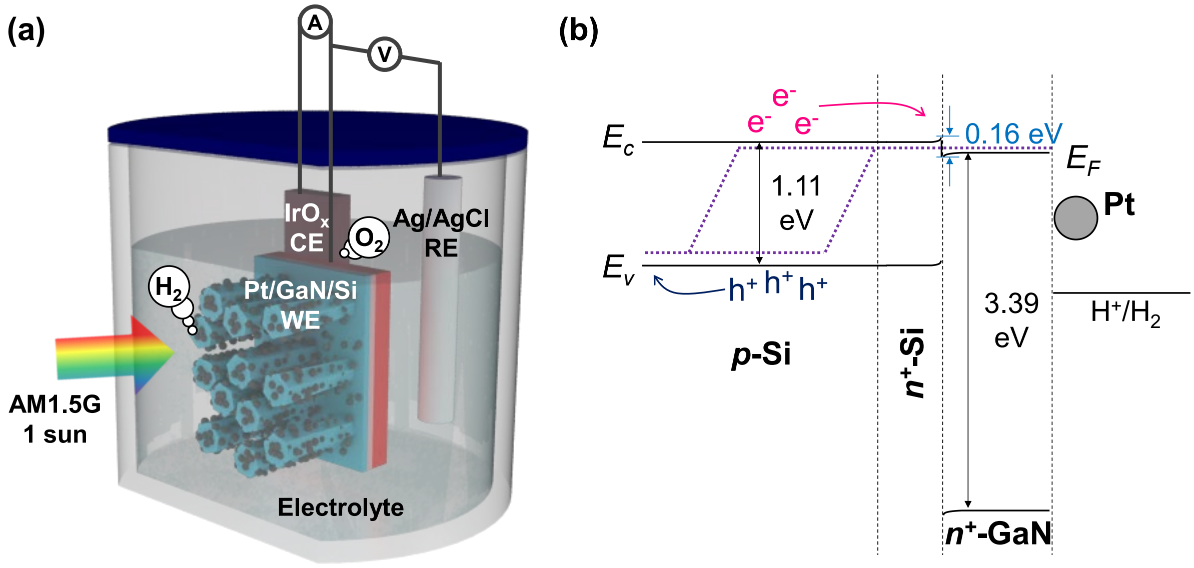

Supplementary Fig. 3. (a) Schematic illustration of reactor for photoelectrochemical hydrogen evolution reaction. IrO_x counter electrode and Ag/AgCl reference electrode were used for measurement with 3-electrode configuration. (b) Schematic of the energy band diagram of Pt/GaN/Si photocathode under light illumination. E_C refers to the conduction band minimum, E_v refers to the valance band maximum, and E_F is fermi level. The energy level of each layer was obtained from literautrue.[1, 2] When light is illuminated on the photocathode, photoelectrons generated in p-Si region spontaneously migrate to n^+-Si and n^+-GaN without significant energy barrier since conduction bands of n^+-Si and n^+-GaN are approximately aligned.

Electrochemical surface area

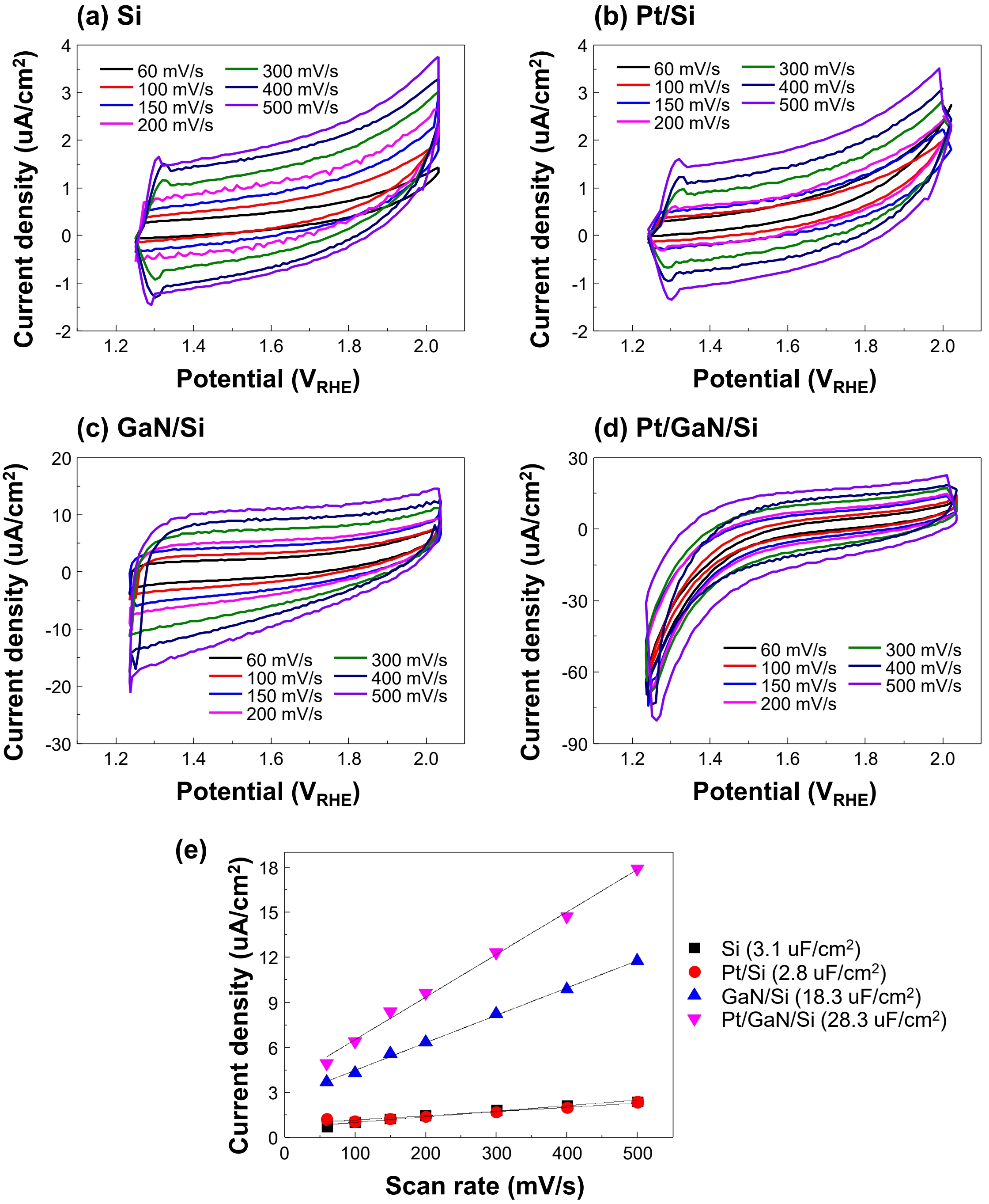

Supplementary Fig. 4. Cyclic voltammetry curves of (a) Si, (b) Pt/Si, (c) GaN/Si, and (d) Pt/GaN/Si. (e) Current density plots at various scan rates. Double layer capacitance, which linearly correlates to electrochemical surface area (ECSA), can be obtained from the slopes of current density-scan rate curves. ECSA of Pt/GaN/Si was ~10 times larger than Pt/Si.

BET surface area

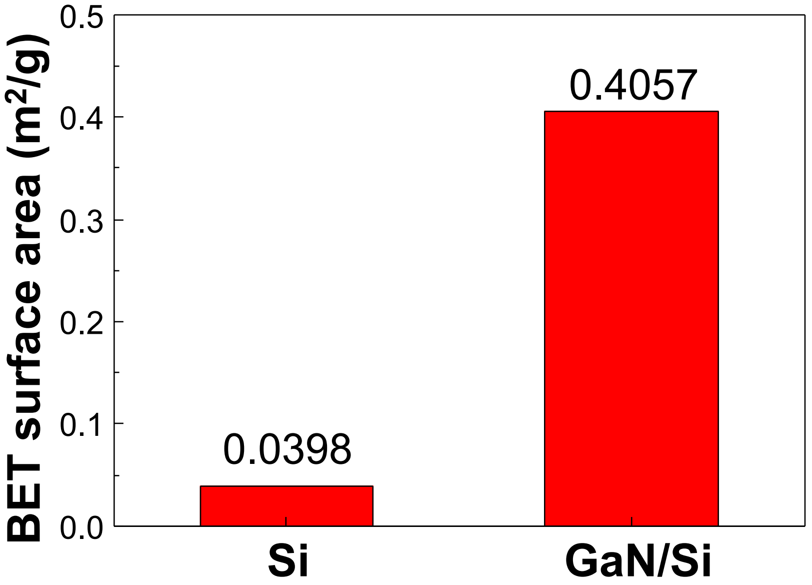

Supplementary Fig. 5. Specific surface area (BET) of Si and GaN/Si measured by adsorption of N_2. BET surface area of GaN/Si was ~10 times larger than Si. This is consistent with ECSA results.

Photoelectrochemical hydrogen evolution reaction in different solutions

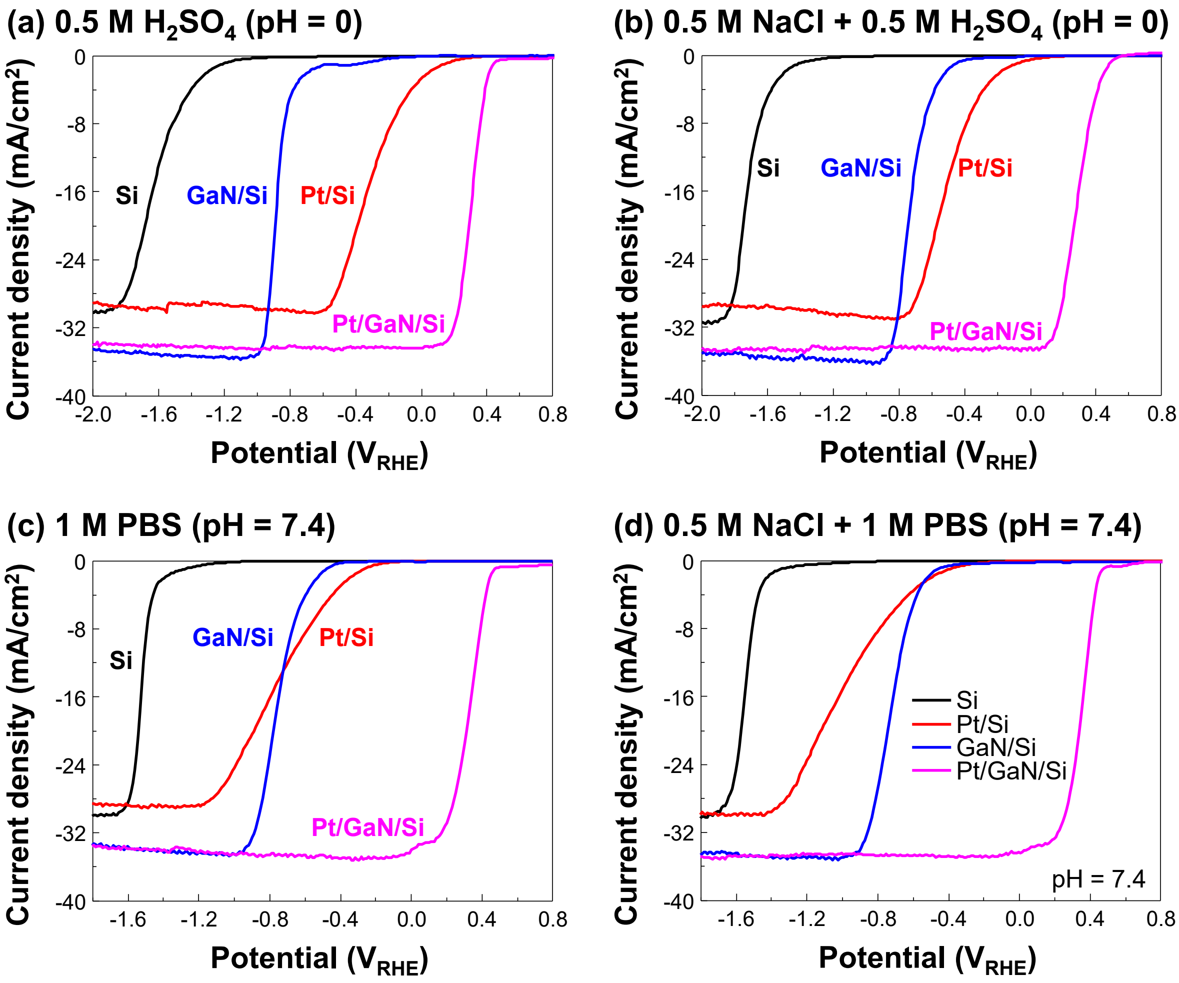

Supplementary Fig. 6. Linear sweep voltammetry (LSV) curves of Si, GaN/Si, Pt/Si, and Pt/GaN/Si in (a) 0.5 M H_2SO_4 (pH = 0), (b) 0.5 M NaCl + 0.5 M H_2SO_4 (pH = 0), (c) 1 M phosphate-buffered solution (PBS) (pH = 7.4), and (d) 0.5 M NaCl + 1 M PBS. The measurements were conducted with a 3-electrode configuration under AM1.5G 1 sun light.

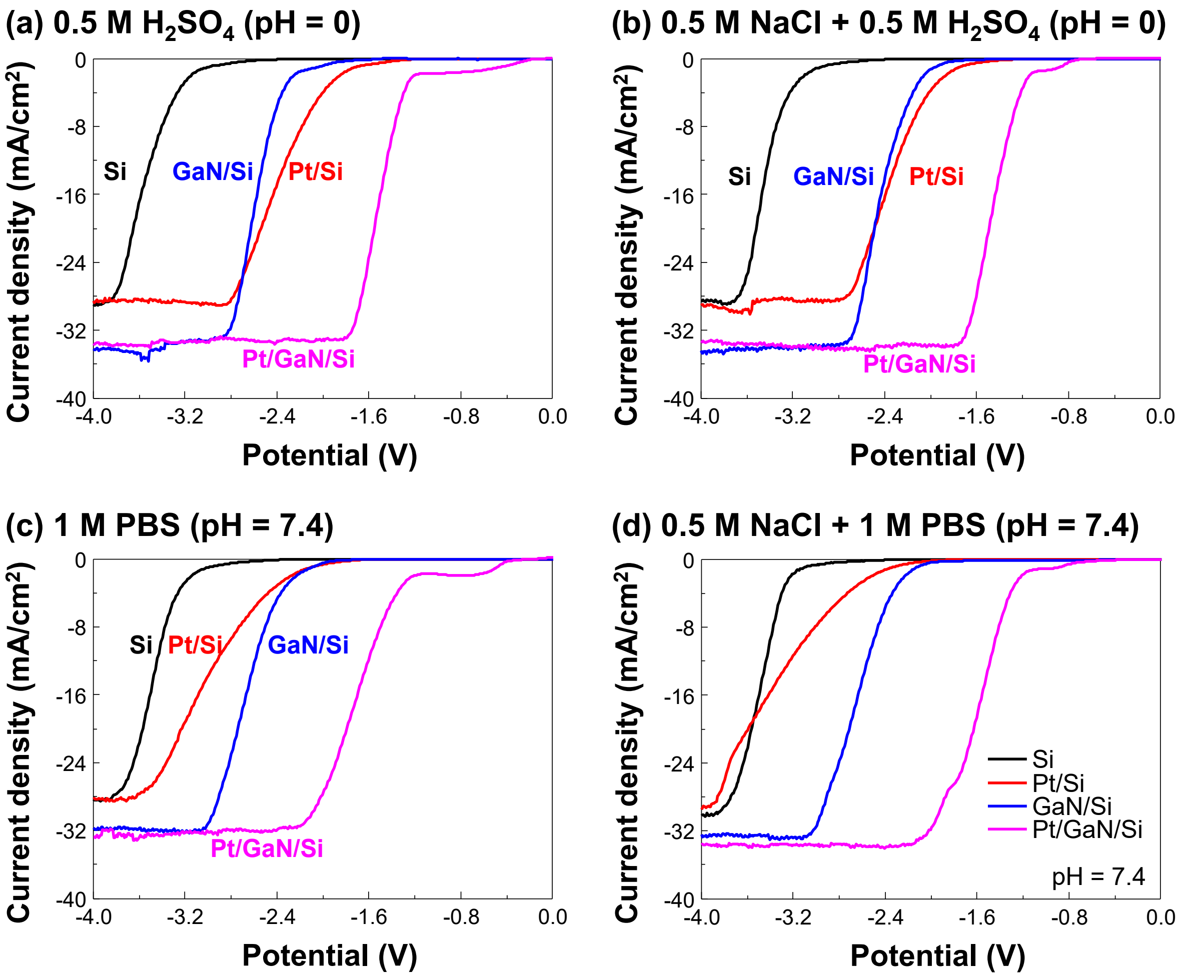

Supplementary Fig. 7. LSV curves of Si, GaN/Si, Pt/Si, and Pt/GaN/Si in (a) 0.5 M H_2SO_4 (pH = 0), (b) 0.5 M NaCl + 0.5 M H_2SO_4 (pH = 0), (c) 1 M PBS (pH = 7.4), and (d) 0.5 M NaCl + 1 M PBS. The measurements were conducted with a 2-electrode configuration under AM1.5G 1 sun light.

PEC HER in NaCl solutions with different molar concentrations

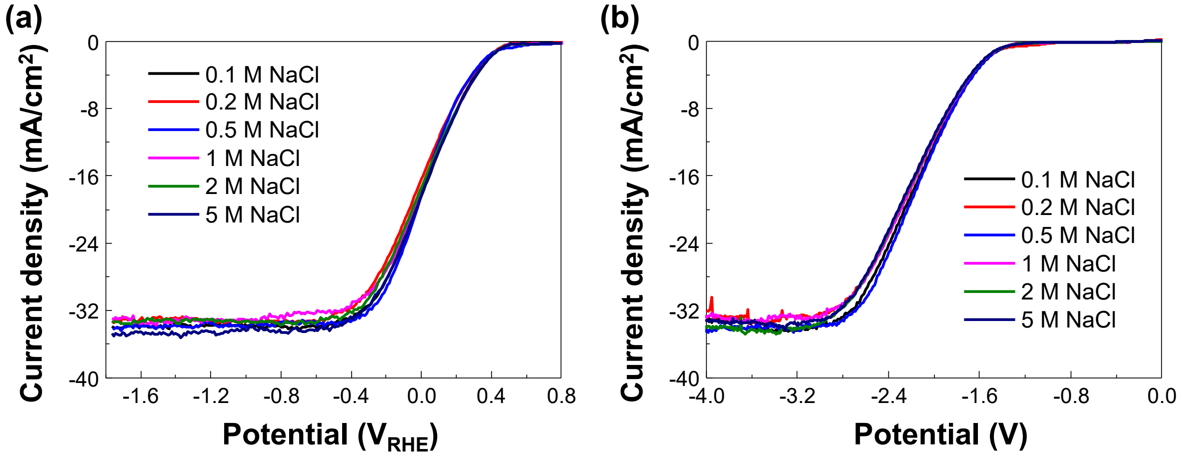

Supplementary Fig. 8. LSV curves of Pt/GaN/Si in NaCl solutions with different molar concentrations (0.1 – 5 M). The measurements were conducted with (a) 3-electrode and (b) 2-electrode configurations under AM1.5G 1 sun light. The photoelectrochemical (PEC) hydrogen evolution reaction (HER) performance was nearly the same regardless of the molar concentration of NaCl.

PEC HER in NaCl + PBS solutions with different molar concentrations of NaCl

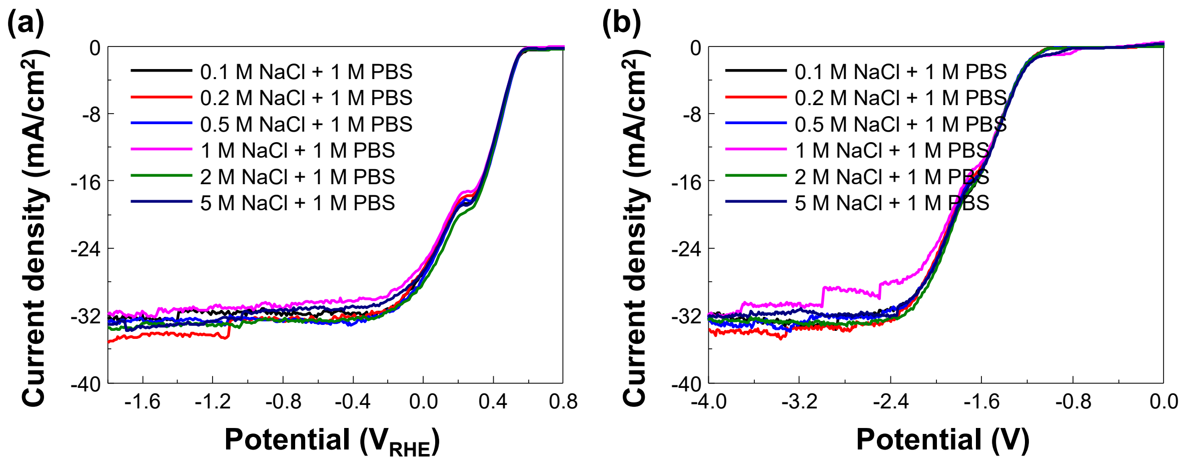

Supplementary Fig. 9. LSV curves of Pt/GaN/Si in NaCl + 1 M PBS solutions with different molar concentrations of NaCl (0.1 – 5 M). The measurements were conducted with (a) 3-electrode and (b) 2-electrode configurations under AM1.5G 1 sun light. The photoelectrochemical (PEC) hydrogen evolution reaction (HER) performance was nearly the same regardless of the molar concentration of NaCl in 1 M PBS solution.

Product analysis

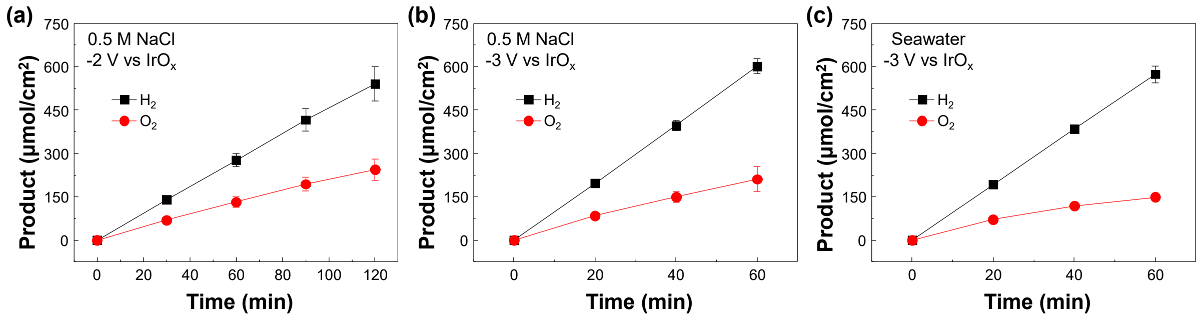

Supplementary Fig. 10. Amount of H_2 and O_2 produced during hydrogen evolution reaction in 0.5 M NaCl at (a) -2 V and (b) -3 V *vs* IrO_x, and (c) in seawater at -3 V *vs* IrO_x. The measurements were conducted 3 times and statistic errors were indicated.

Stability test in 0.5 M NaCl

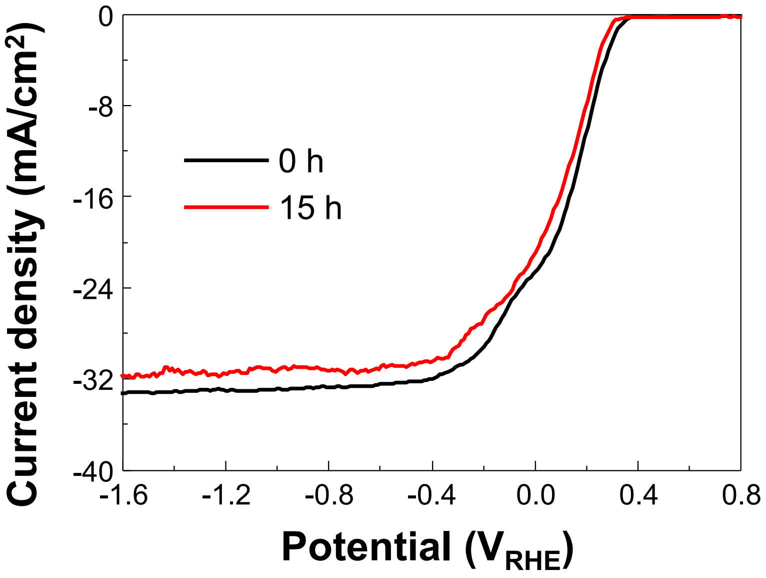

Supplementary Fig. 11. LSV curves of Pt/GaN/Si photocathode in 0.5 M NaCl before and after the stability test for 15 h at – 3 V *vs* IrO_x.

Stability test in seawater

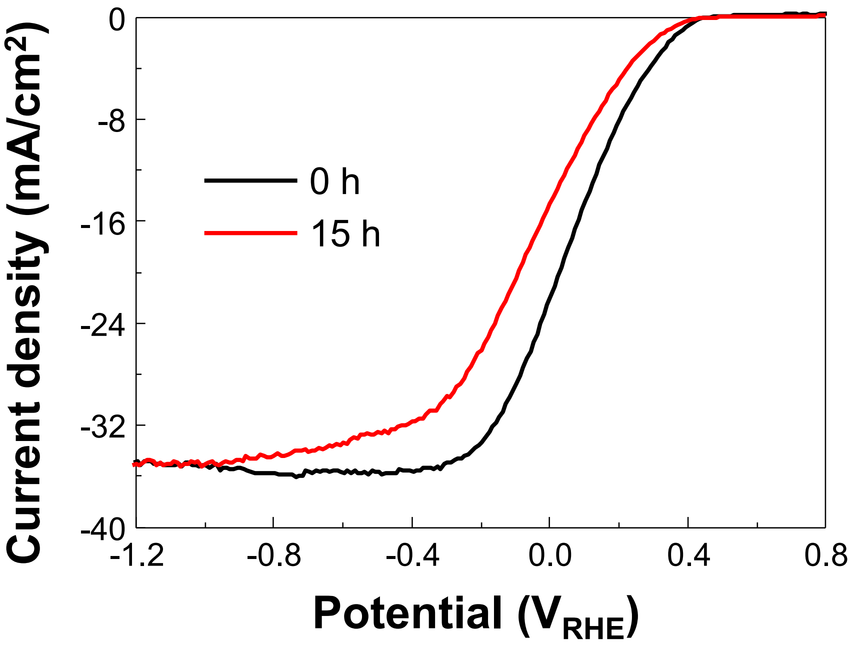

Supplementary Fig. 12. LSV curves of Pt/GaN/Si photocathode in seawater before and after the stability test for 15 h at – 3 V *vs* IrO_x.

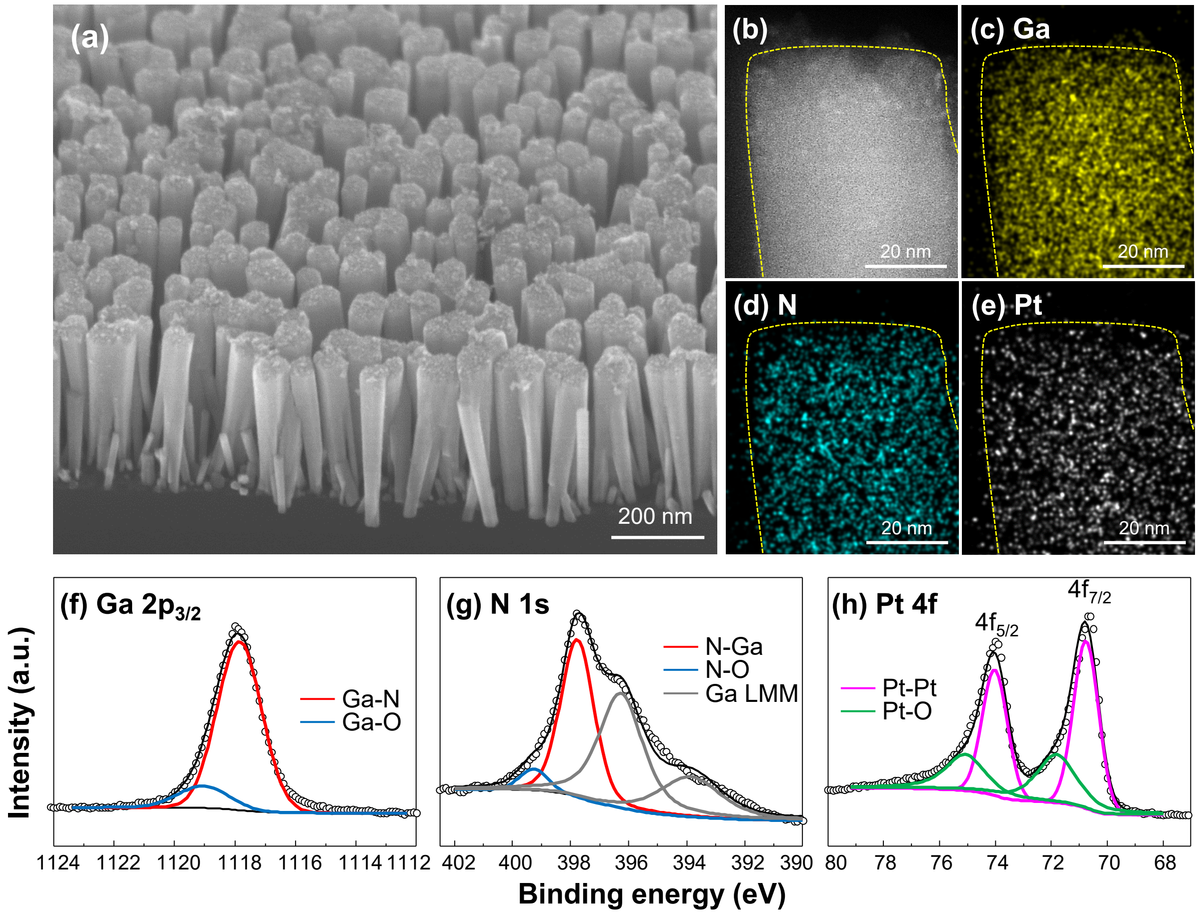

Supplementary Fig. 13. Pt/GaN/Si after the stability test in 0.5 M NaCl for 15 h at -3 V *vs* IrO_x. (a) Tilt-view SEM image, (b) HAADF-STEM image, STEM-EDS elemental maps of (c) Ga, (d) N, (e) Pt, and XPS spectra of (a) Ga *2p3/2*, (g) N *1s*, and (h) Pt *4f*. The morphology and composition of Pt/GaN/Si remains almost identical even after long-term stability test. However, the atomic ratio of Pt/(Ga+N) calculated from XPS analysis decreased from 0.042 to 0.037 after 15 h of reaction.

Stability test in 0.5 M NaCl + 1 M PBS

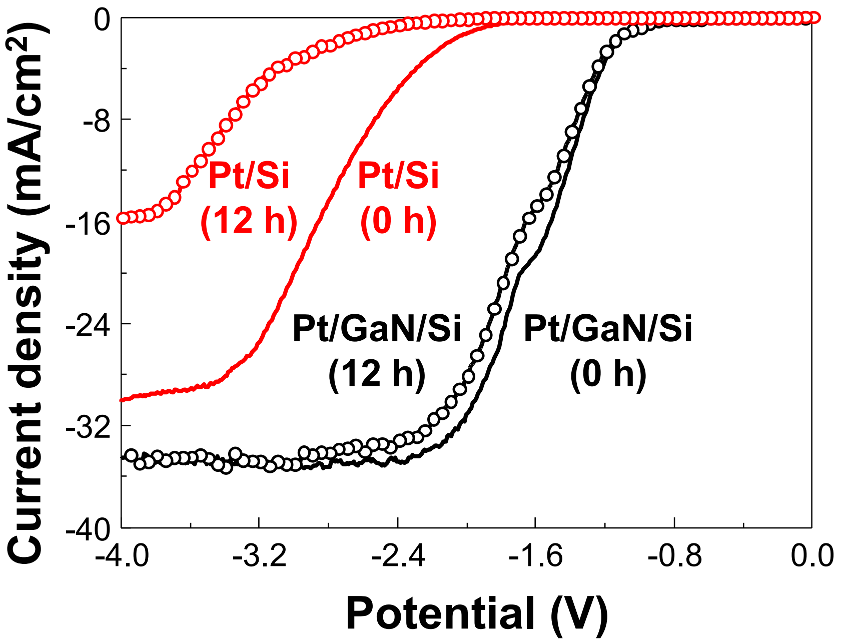

Supplementary Fig. 14. LSV curves of Pt/Si and Pt/GaN/Si in 0.5 M NaCl + 1 M PBS before and after the stability test for 12 h at -2.5 V *vs* IrO_x. The measurements were conducted with 2-electrode configuration under AM1.5G 1 sun light.

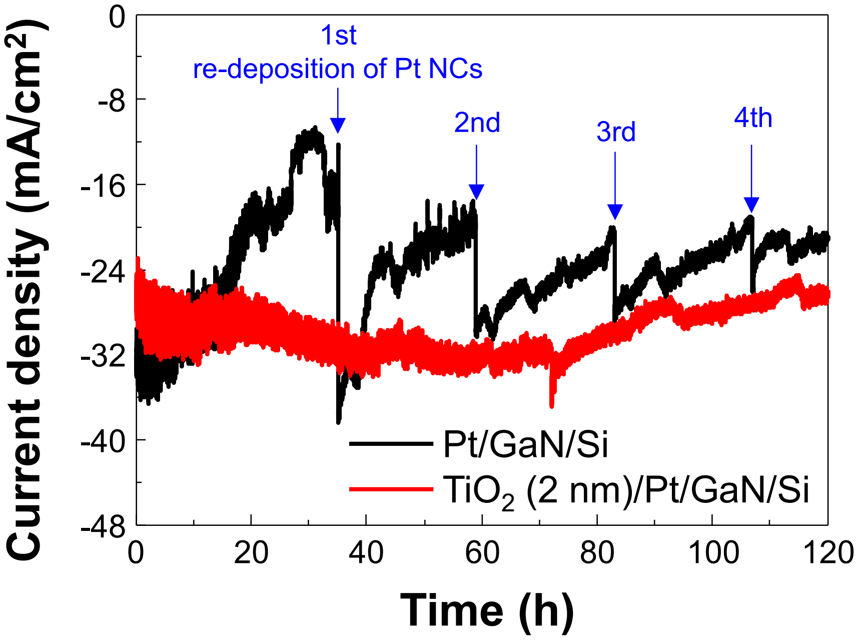

Supplementary Fig. 15. Long-term stability of Pt/GaN/Si (black curve) and TiO_2/Pt/GaN/Si (red curve) at -3 V (*vs* IrO_x) in 0.5 M NaCl under 1-sun light illumination. Pt/GaN/Si degraded after 18 h of reaction. After the re-deposition of Pt NCs, the photocurrent density recovered. 2 nm-thick TiO_2 passivation layer effectively stabilized the photocurrent density ~30 mA/cm^2 for 120 h, indicating that Pt NCs were more strongly anchored on GaN NWs by a TiO_2 passivation layer.

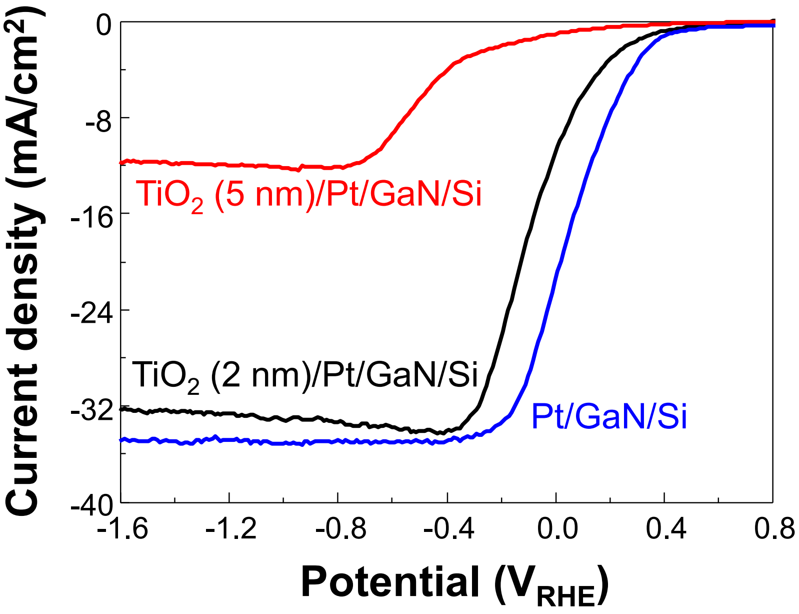

Supplementary Fig. 16. LSV curves of Pt/GaN/Si and TiO_2/Pt/GaN/Si photocathodes measured with 3-electrode configuration in 0.5 M NaCl solution. When the thickness of TiO_2 layer on Pt/GaN/Si increased to 2 and 5 nm, the onset potential at -10 mA/cm^2 was negatively shifted from 0.16 V_{RHE} to 0.01 V_{RHE} and -0.62 V_{RHE}, respectively. The photocurrent density also gradually decreased as the thickness of TiO_2 increased. The decrease in performance is likely due to the shielding of Pt-Ga sites by inactive and electrically resistive TiO_2 layer.

Slab models for DFT calculation

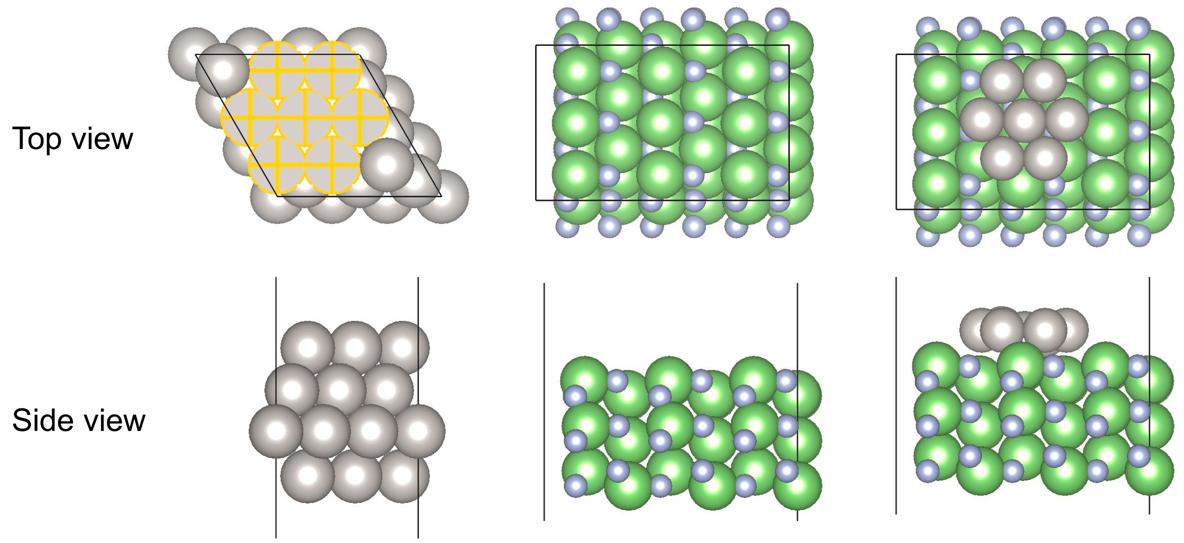

Supplementary Fig. 17. Slab models of Pt(111), GaN$(10\bar{1}0)$, and Pt_7@GaN$(10\bar{1}0)$. The Pt_7 cluster was taken from the Pt(111) surface (highlighted atoms). The blue, green, and grey spheres represent N, Ga, and Pt atoms, respectively.

Water dissociation at Pt/GaN and Pt/GaON interface

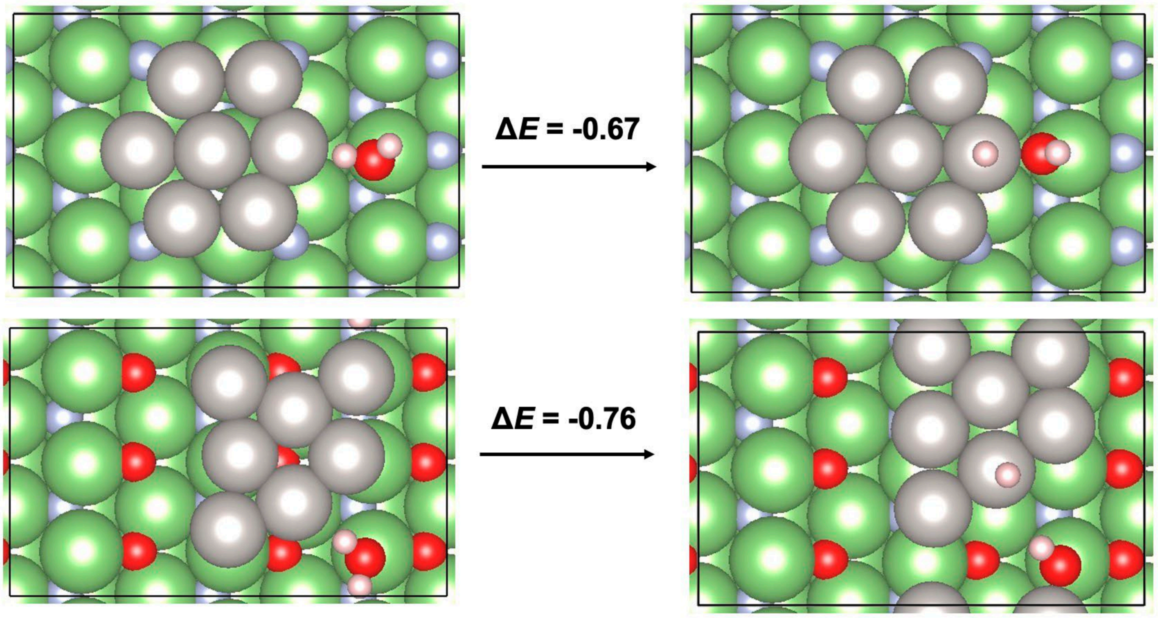

Supplementary Fig. 18. Energy changes of water dissociation at the Pt/GaN (upper panel) and Pt/GaON (lower panel) interfaces (in eV). The blue, red, green, and grey spheres represent N, O, Ga, and Pt atoms, respectively. The black box indicated the supercell we used in our calculations.

PEC HER under the concentrated light (9 suns) in 0.5 M NaCl

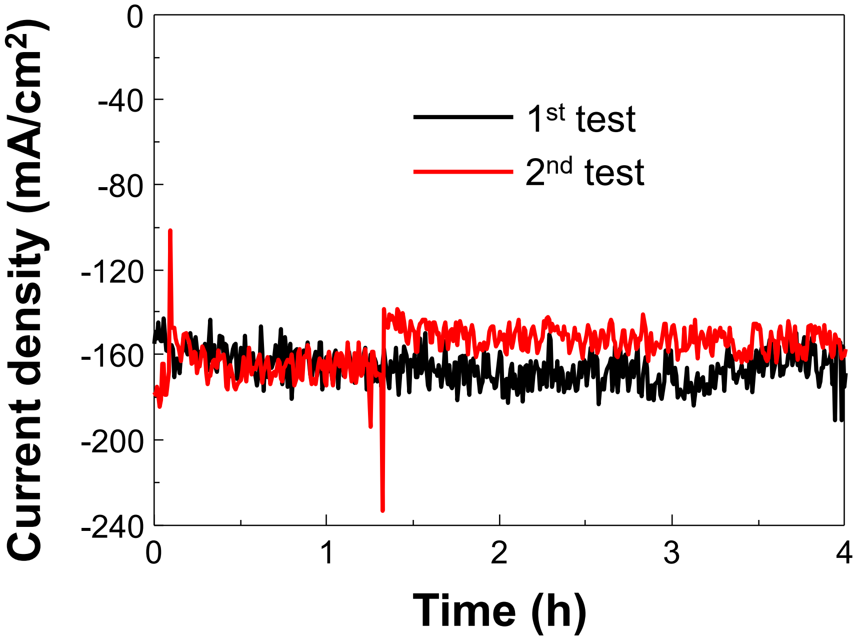

Supplementary Fig. 19. Chronoamperometric curves of two individual Pt/GaN/Si photocathodes measured under light intensity of 9 suns at -3 V vs IrO_x in 0.5 M NaCl. There was no noticeable degradation for 4 h reaction.

PEC HER under the concentrated light in 0.5 M NaCl + 1 M PBS

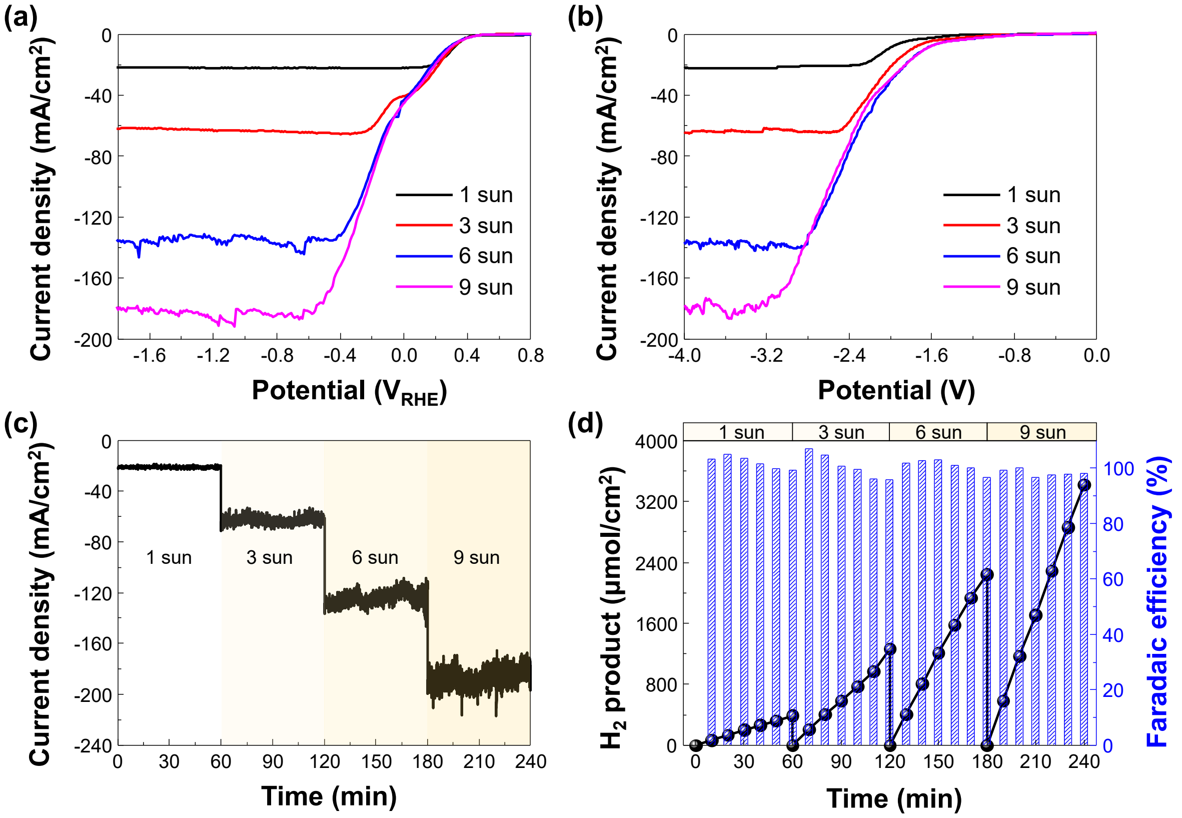

Supplementary Fig. 20. LSV curves of Pt/GaN/Si measured with (a) 3-electrode and (b) 2-electrode configurations in 0.5 M NaCl + 1 M PBS under different light intensities. (c) Chronoamperometric curve and (d) amount of H_2 produced and faradaic efficiency measured under light intensities of 1, 3, 6, and 9 suns at -3 V *vs* IrO_x.

Electrochemical HER under dark condition

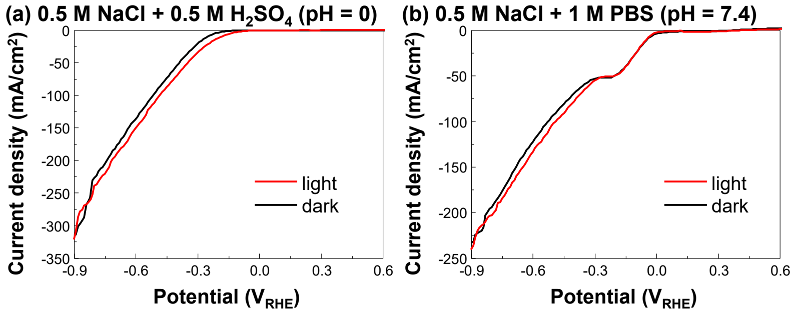

Supplementary Fig. 21. LSV curves of front contact electrochemical HER measured with 3-electrode configuration in (a) 0.5 M NaCl + 0.5 M H_2SO_4, (b) 0.5 M NaCl + 1 M PBS, and (c) 0.5 M NaCl solutions.

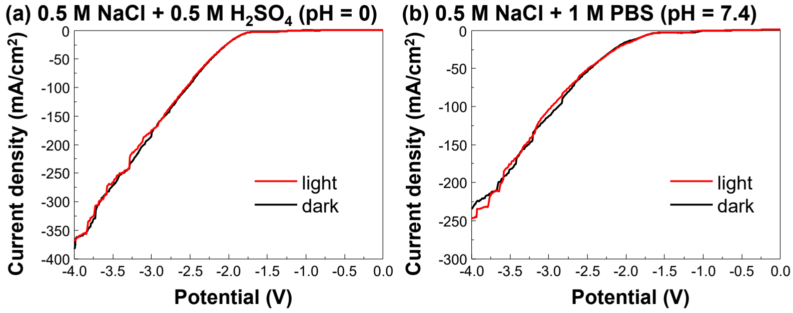

Supplementary Fig. 22. LSV curves of front contact electrochemical HER measured with 2-electrode configuration in (a) 0.5 M NaCl + 0.5 M H_2SO_4, (b) 0.5 M NaCl + 1 M PBS, and (c) 0.5 M NaCl solutions.

Switchable photo/electrochemical HER under light and dark conditions

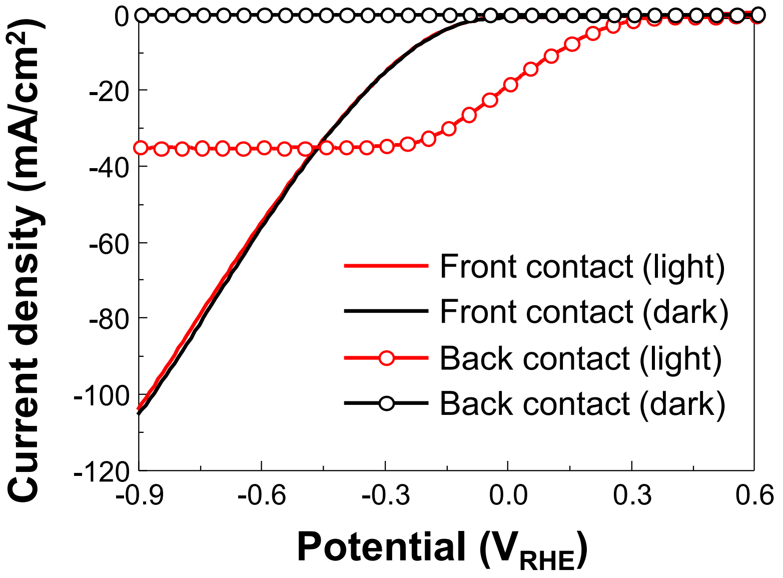

Supplementary Fig. 23. (a) LSV curves of dual contact electrode measured with 3-electrode configuration in 0.5 M NaCl solution.

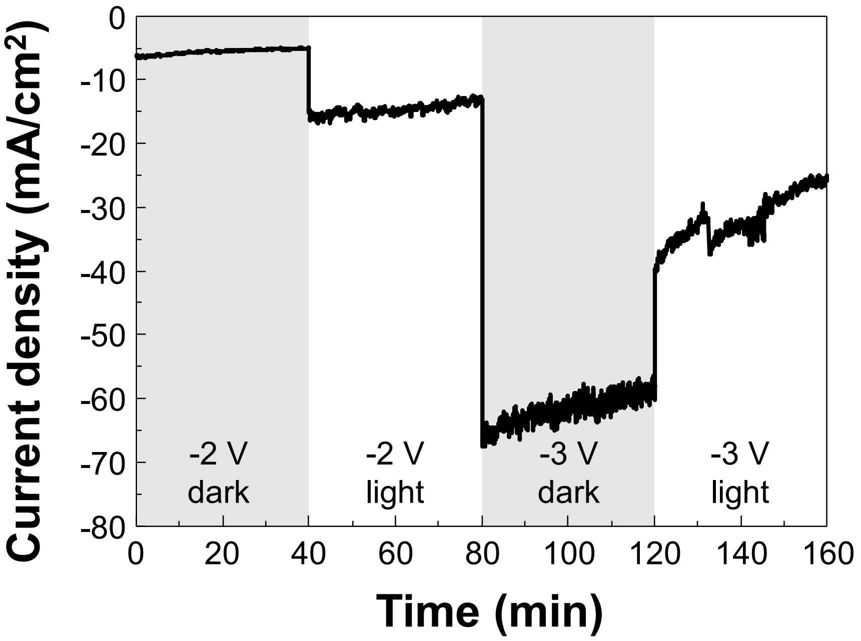

Supplementary Fig. 24. Chronoamperometric curve at -2 and -3 V under dark (front contact) and light (back contact) conditions. The measurement was conducted with 2-electrode configuration.

Supplementary Table 1. Performance and reaction condition comparison of catalysts for hydrogen evolution reaction in seawater from recent literature.

Cathodes	Electrolyte (pH)	η_{10} (3-electrode configuration)	2-electrode cathode || anode	η_{10} (2-electrode configuration)	Ref.
Pt/GaN/Si	0.5 M NaCl (9.1)	0.16 V_{RHE} (PEC)<br>-0.40 V_{RHE} (EC)	Pt/GaN/Si || IrO_x	-1.88 V (PEC)<br>-2.33 V (EC)	This work
	0.5 M NaCl + 1 M PBS (7.4)	0.40 V_{RHE} (PEC)<br>-0.05 V_{RHE} (EC)	Pt/GaN/Si || IrO_x	-1.45 V (PEC)<br>-1.83 V (EC)	
p-Si/TiO_2/NiO_x	Artificial seawater (8.4)	~-0.7 V_{RHE} (PEC)	NA		3
Co_3O_4	Natural seawater (7.69)	~-0.6 V_{RHE} (PEC)	NA		4
Ni_2P-Fe_2P	1 M KOH seawater (~14)	η_{100} = -0.252 V_{RHE}	Ni_2P-Fe_2P || Ni_2P-Fe_2P	η_{100} = -1.811 V	5
Ru/WNO@C nanowires	3 M NaOH + 3 M NaCl (~14)	-0.0269 V_{RHE}	Ru/WNO@C || RuO_2/IrO_2-coated Ti-mesh	-2.48 V	6
Ru-CoO_x/Ni foam	1 M KOH + seawater (~14)	~-0.02 V_{RHE}	Ru-CoO_x/NF || Ru-CoO_x/NF	-1.86 V	7
Co-Fe_2P	1 M KOH + seawater (~14)	η_{100} = -0.221 V_{RHE}	Co-Fe_2P || Co-Fe_2P	η_{100} = -1.69 V	8
NiMoN@NiFeN	1 M KOH + seawater (~14)	η_{100} = -0.082 V_{RHE}	NiMoN || NiMoN@NiFeN	η_{100} = -1.581 V	9
NiCoN|Ni_xP|NiCoN microsheet	Seawater (7.2)	-0.165 V_{RHE}	NiCoN|Ni_xP|NiCoN || S-(Ni,Fe)OOH	-1.81 V	10
Ni surface nitride (Ni-SN@C)	1 M KOH seawater (~14)	-0.023 V_{RHE}	Ni-SN@C || Ni-SN@C	-1.72 V	11
NiCoP nanoarrays	Seawater (8.4)	-0.287 V_{RHE}	NA		12
Mo_5N_6 nanosheet	Seawater (8.4)	-0.257 V_{RHE}	NA		13
Rh with N/S-doped C	Seawater (8.32)	-0.340 V_{RHE}	NA		14

$Co_{0.31}Mo_{1.69}C$/MXene/NC	Seawater (8.28)	-0.208 V_{RHE}	NA	15
Pt/Ni-Mo	1 M KOH + 0.5 M NaCl (~14)	η_{2000} = -0.113 V_{RHE}	NA	16
Pt@multi-level hollow structures Mxene	Seawater (8.28)	-0.280 V_{RHE}	NA	17
h-MoN@BNCNT	Seawater (7.9)	~-0.16 V_{RHE}	NA	18
Ni single atom/NC	1 M KOH + seawater (13.8)	-0.139 V_{RHE}	NA	19
Mo@(2H-1T)-$MoSe_2$	Seawater (8.26)	η_{20} = -0.470 V_{RHE}	NA	20
Rh/N-doped hollow carbon spheres	Seawater (8.35)	-0.220 V_{RHE}	NA	21
Graphdiyne/MoO_3	Seawater (8.21)	~-0.3 V_{RHE}	NA	22
Rh_2P/NPC	Seawater (7.8)	-0.160 V_{RHE}	NA	23
VS_2@V_2C	Seawater (8.6)	~-0.15 V_{RHE}	NA	24
CoNiP/Co_xP	Seawater (8.19)	-0.290 V_{RHE}	NA	25

Supplementary Table 2. Performance and reaction condition comparison of Si-based photocathodes for hydrogen evolution reaction from literature.

Photocathodes	Electrolyte (pH)	Light intensity	Potential	Current density	ABPE	Ref.
Pt/GaN/n^+p Si	0.5 M NaCl (9.1)	1 sun	0.16 V_{RHE} at 10 mA/cm^2	21.6 mA/cm^2 at 0 V_{RHE}	1.6%	**This work**
	0.5 M NaCl + 1 M PBS (7.4)	1 sun	0.40 V_{RHE} at 10 mA/cm^2	34.3 mA/cm^2 at 0 V_{RHE}	7.9%	
	0.5 M NaCl (9.1)	9 sun	0.23 V_{RHE} at 10 mA/cm^2	165 mA/cm^2 at -0.6 V_{RHE}	-	
	0.5 M NaCl + 1 M PBS (7.4)	9 sun	0.26 V_{RHE} at 10 mA/cm^2	182 mA/cm^2 at -0.6 V_{RHE}	-	
Pt/TiO_2/InAs NWs/p-Si	PBS with 0.5 M Na_2SO_4 (7.0)	1 sun	0.48 V_{RHE} at 1 mA/cm^2	8.6 mA/cm^2 at 0 V_{RHE}	1.9%	[26]
Pt/TiO_2/CdS/p-Si	PBS (6.8)	1 sun	0.42 V_{RHE}	21.9 mA/cm^2 at 0 V_{RHE}	2.07%	[27]
NiO_x-Fe_2O_3/SnO_2/p-Si	PBS with 0.25 M Na_2SO_4 (7.1)	1 sun	0.25 V_{RHE} at 0.1 mA/cm^2	0.25 mA/cm^2 at 0 V_{RHE}	NA	[28]
NiO_x/TiO_2/p-Si microwires	PBS (7.0)	1 sun	0.42 V_{RHE} at 0.1 mA/cm^2	1.48mA/cm^2 at 0 V_{RHE}	1.74%	[3]
n-ZnO/p-Si NWs	0.25 M Na_2SO_4 (7.2)	1 sun	-0.5 $V_{Ag/AgCl}$	~6 mA/cm^2 at -1.5 $V_{Ag/AgCl}$	NA	[29]
Fe_2O_3/SnO_2/p-Si NWs	0.25 M Na_2SO_4 (7.2)	1 sun	-0.55 V_{RHE}	~2 mA/cm^2 at -0.75 V_{RHE}	NA	[30]
g-C_3N_4/p-Si NWs	0.5 M Na_2SO_4 (~7)	1 sun	0.42 V_{RHE}	0.5 mA/cm^2 at 0 V_{RHE}	4.3%	[31]
g-C_3N_4 NSs-$SrTiO_3$ NPs/p-Si NWs	0.5 M Na_2SO_4 (~7)	1 sun	0.606 V_{RHE}	0.97 mA/cm^2 at 0 V_{RHE}	5.4%	[32]
$CoSe_2$/p-Si NWs	1 M PBS (6.5)	1 sun	0.137 V_{RHE} at 1 mA/cm^2	2.6 mA/cm^2 at 0 V_{RHE}	NA	[33]
NiP_2/Ti/n^+p-Si	1 M KBi buffer (9.5)	1 sun	0.35 V_{RHE}	Saturation J = 19 mA/cm^2	2.6%	[34]

NiFe LDH/Ti/p-Si	1 M KOH (14)	1 sun	~0.3 V_{RHE} at 1 mA/cm^2	7 mA/cm^2 at 0 V_{RHE}	NA	[35]
Pt/TiO_2/n^+np^+-Si	1 M $HClO_4$ (0.0)	1 sun	0.52 V_{RHE} at 1 mA/cm^2	~35.1 mA/cm^2 at 0 V_{RHE}	11.5%	[36]
MoS_2/TiO_2/p-Si	0.5 M H_2SO_4 (0.0)	1 sun	0.35 V_{RHE} at 1 mA/cm^2	28 mA/cm^2 at 0 V_{RHE}	1.8%	[37]
$MoSe_2$/n^+p-Si	1 M $HClO_4$ (0.0)	1 sun	0.4 V_{RHE} at 1 mA/cm^2	Saturation J = 29.3 mA/cm^2	3.8%	[38]
Pt/n^+p-Si	1 M $HClO_4$ (0.0)	1 sun	0.56 V_{RHE} at 1 mA/cm^2	Saturation J = 34.6 mA/cm^2	10.8%	[39]
MoS_2/Al_2O_3/n^+p-Si	1 M $HClO_4$ (0.0)	1 sun	0.4 V_{RHE} at 1 mA/cm^2	Saturation J = 35.6 mA/cm^2	NA	[40]
Ni-Mo/n^+p-Si microwires	KHP with KOH (4.5)	1 sun	0.46 V_{RHE} at 1 mA/cm^2	9.1 mA/cm^2 at 0 V_{RHE}	1.9%	[41]
MoS_2/n^+p-Si	0.5 M H_2SO_4 (0.0)	1 sun	0.32 V_{RHE} at 0.5 mA/cm^2	Saturation J ~ 17 mA/cm^2	NA	[42]
Pt/Ti/$SrTiO_3$/p-Si	0.5 M H_2SO_4 (0.0)	1 sun	0.46 V_{RHE}	Saturation J ~ 35 mA/cm^2	4.9%	[43]
Pt/TiO_2 nanorods/p-Si	0.5 M H_2SO_4 (0.0)	1 sun	0.44 V_{RHE}	40 mA/cm^2 at 0 V_{RHE}	2.5%	[44]
$NiCoSe_c$/p-Si nanopillar	0.5 M H_2SO_4 (0.0)	1 sun	0.32 V_{RHE} at 0.1 mA/cm^2	37.5 mA/cm^2 at 0 V_{RHE}	NA	[45]

References

1. Vanka S*, et al.* High efficiency Si photocathode protected by multifunctional GaN nanostructures. *Nano Lett.* **18**, 6530-6537 (2018).

2. Wu Y*, et al.* III-Nitride Nanostructures: Emerging Applications for Micro-LEDs, Ultraviolet Photonics, Quantum Optoelectronics, and Artificial Photosynthesis. *Prog. Quantum Electron.*, 100401 (2022).

3. Kawde A*, et al.* Photo-electrochemical hydrogen production from neutral phosphate buffer and seawater using micro-structured p-Si photo-electrodes functionalized by solution-based methods. *Sustain. Energy Fuels* **2**, 2215-2223 (2018).

4. Patel M, Park W-H, Ray A, Kim J, Lee J-H. Photoelectrocatalytic sea water splitting using Kirkendall diffusion grown functional Co_3O_4 film. *Sol. Energy Mater. Sol. Cells* **171**, 267-274 (2017).

5. Wu L*, et al.* Heterogeneous bimetallic phosphide Ni_2P-Fe_2P as an efficient bifunctional catalyst for water/seawater splitting. *Adv.*

Funct. Mater. **31**, 2006484 (2021).

6. Zhang L-N, *et al.* Cable-like Ru/WNO@C nanowires for simultaneous high-efficiency hydrogen evolution and low-energy consumption chlor-alkali electrolysis. *Energy Environ. Sci.* **12**, 2569-2580 (2019).

7. Wu D, Chen D, Zhu J, Mu S. Ultralow Ru Incorporated Amorphous Cobalt-Based Oxides for High-Current-Density Overall Water Splitting in Alkaline and Seawater Media. *Small* **17**, 2102777 (2021).

8. Wang S, *et al.* Synthesis of 3D heterostructure Co-doped Fe_2P electrocatalyst for overall seawater electrolysis. *Appl. Catal. B* **297**, 120386 (2021).

9. Yu L, *et al.* Non-noble metal-nitride based electrocatalysts for high-performance alkaline seawater electrolysis. *Nat. Commun.* **10**, 1-10 (2019).

10. Yu L, *et al.* Hydrogen Generation from Seawater Electrolysis over a Sandwich-like NiCoN| Ni x P| NiCoN Microsheet Array Catalyst. *ACS Energy Lett.* **5**, 2681-2689 (2020).

11. Jin H, *et al.* Stable and highly efficient hydrogen evolution from seawater enabled by an unsaturated nickel surface nitride. *Adv. Mater.* **33**, 2007508 (2021).

12. Lv Q, Han J, Tan X, Wang W, Cao L, Dong B. Featherlike NiCoP holey nanoarrys for efficient and stable seawater splitting. *ACS Appl. Energy Mater.* **2**, 3910-3917 (2019).

13. Jin H, *et al.* Single-crystal nitrogen-rich two-dimensional Mo_5N_6 nanosheets for efficient and stable seawater splitting. *ACS Nano* **12**, 12761-12769 (2018).

14. Liu Y, Hu X, Huang B, Xie Z. Surface engineering of Rh catalysts with N/S-codoped carbon nanosheets toward high-Performance hydrogen evolution from seawater. *ACS Sustain. Chem. Eng.* **7**, 18835-18843 (2019).

15. Wu X, *et al.* Engineering multifunctional collaborative catalytic interface enabling efficient hydrogen evolution in all pH range and seawater. *Adv. Energy Mater.* **9**, 1901333 (2019).

16. Yang F, *et al.* A durable and efficient electrocatalyst for saline water splitting with current density exceeding 2000 mA cm^{-2}. *Adv. Funct. Mater.* **31**, 2010367 (2021).

17. Xiu L, *et al.* Multilevel hollow MXene tailored low-Pt catalyst for efficient hydrogen evolution in full-pH range and seawater. *Adv. Funct. Mater.* **30**, 1910028 (2020).

18. Miao J, *et al.* Polyoxometalate-derived hexagonal molybdenum nitrides (MXenes) supported by boron, nitrogen codoped carbon nanotubes for efficient electrochemical hydrogen evolution from seawater. *Adv. Funct. Mater.* **29**, 1805893 (2019).

19. Zang W, *et al.* Efficient Hydrogen Evolution of Oxidized Ni-N_3 Defective Sites for Alkaline Freshwater and Seawater Electrolysis. *Adv. Mater.* **33**, 2003846 (2021).

20. Yang C, *et al.* Large-scale synthetic Mo@(2H-1T)-$MoSe_2$ monolithic electrode for efficient hydrogen evolution in all pH scale ranges and seawater. *Appl. Catal. B* **304**, 120993 (2022).

21. Ding R, Yan T, Wang Y, Long Y, Fan G. Carbon nanopore and anchoring site-assisted general construction of encapsulated metal (Rh, Ru, Ir) nanoclusters for highly efficient hydrogen evolution in pH-universal electrolytes and natural seawater. *Green Chem.* **23**, 4551-4559 (2021).

22. Yao Y, *et al.* Interfacial sp C–O–Mo hybridization originated high-current density hydrogen evolution. *J. Am. Chem. Soc.* **143**, 8720-8730 (2021).

23. Liu S, *et al.* A supramolecular-confinement pyrolysis route to ultrasmall rhodium phosphide nanoparticles as a robust electrocatalyst for hydrogen evolution in the entire pH range and seawater electrolysis. *J. Mater. Chem. A* **8**, 25768-25779 (2020).

24. Wang Z, Xu W, Yu K, Feng Y, Zhu Z. 2D heterogeneous vanadium compound interfacial modulation enhanced synergistic catalytic hydrogen evolution for full pH range seawater splitting. *Nanoscale* **12**, 6176-6187 (2020).

25. Liu D, *et al.* Multi-Phase Heterostructure of CoNiP/Co_xP for Enhanced Hydrogen Evolution Under Alkaline and Seawater Conditions by Promoting H_2O Dissociation. *Small* **17**, 2007557 (2021).

26. Choi S, *et al.* Photoelectrochemical hydrogen production at neutral pH phosphate buffer solution using TiO_2 passivated InAs Nanowire/p-Si heterostructure photocathode. *Chem. Eng. J.* **392**, 123688 (2020).

27. Liu S, *et al.* Multifunctional TiO_2 overlayer for p-Si/n-CdS heterojunction photocathode with improved efficiency and stability. *Nano Energy* **53**, 125-129 (2018).

28. Kargar A, *et al.* NiO_x-Fe_2O_3-coated p-Si photocathodes for enhanced solar water splitting in neutral pH water. *Nanoscale* **7**, 4900-4905 (2015).

29. Kargar A, *et al.* Tailoring n-ZnO/p-Si branched nanowire heterostructures for selective photoelectrochemical water oxidation or reduction. *Nano Lett.* **13**, 3017-3022 (2013).

30. Kargar A*, et al.* p-Si/SnO_2/Fe_2O_3 Core/Shell/Shell Nanowire Photocathodes for Neutral pH Water Splitting. *Adv. Funct. Mater.* **25**, 2609-2615 (2015).

31. Gopalakrishnan S, Bhalerao G, Jeganathan K. g-C_3N_4 nanosheets functionalized silicon nanowires hybrid photocathode for efficient visible light induced photoelectrochemical water reduction. *J. Power. Sources* **413**, 293-301 (2019).

32. Gopalakrishnan S, Bhalerao G, Jeganathan K. $SrTiO_3$ NPs/g-C_3N_4 NSs coupled Si NWs based hybrid photocathode for visible light driven photoelectrochemical water reduction. *ACS Sustain. Chem. Eng.* **7**, 13911-13919 (2019).

33. Chen C-J*, et al.* Wide range pH-tolerable silicon@ pyrite cobalt dichalcogenide microwire array photoelectrodes for solar hydrogen evolution. *ACS Appl. Mater. Interfaces* **8**, 5400-5407 (2016).

34. Chen F, Zhu Q, Wang Y, Cui W, Su X, Li Y. Efficient photoelectrochemical hydrogen evolution on silicon photocathodes interfaced with nanostructured NiP_2 cocatalyst films. *ACS Appl. Mater. Interfaces* **8**, 31025-31031 (2016).

35. Zhao J, Cai L, Li H, Shi X, Zheng X. Stabilizing silicon photocathodes by solution-deposited Ni–Fe layered double hydroxide for efficient hydrogen evolution in alkaline media. *ACS Energy Lett.* **2**, 1939-1946 (2017).

36. Yin Z, Fan R, Huang G, Shen M. 11.5% efficiency of TiO_2 protected and Pt catalyzed n^+np^+-Si photocathodes for photoelectrochemical water splitting: manipulating the Pt distribution and Pt/Si contact. *Chem. Commun.* **54**, 543-546 (2018).

37. Andoshe DM*, et al.* Directly assembled 3D molybdenum disulfide on silicon wafer for efficient photoelectrochemical water reduction. *Adv. Sustain. Syst.* **2**, 1700142 (2018).

38. Huang G*, et al.* Integrated $MoSe_2$ with n^+p-Si photocathodes for solar water splitting with high efficiency and stability. *Appl. Phys. Lett.* **112**, 013902 (2018).

39. Fan R, Dong W, Fang L, Zheng F, Shen M. More than 10% efficiency and one-week stability of Si photocathodes for water splitting by manipulating the loading of the Pt catalyst and TiO_2 protective layer. *J. Mater. Chem. A* **5**, 18744-18751 (2017).

40. Fan R*, et al.* Efficient and stable silicon photocathodes coated with vertically standing nano-MoS_2 films for solar hydrogen production. *ACS Appl. Mater. Interfaces* **9**, 6123-6129 (2017).

41. Warren EL, McKone JR, Atwater HA, Gray HB, Lewis NS. Hydrogen-evolution characteristics of Ni–Mo-coated, radial junction, n^+p-silicon microwire array photocathodes. *Energy Environ. Sci.* **5**, 9653-9661 (2012).

42. Benck JD, Lee SC, Fong KD, Kibsgaard J, Sinclair R, Jaramillo TF. Designing active and stable silicon photocathodes for solar

hydrogen production using molybdenum sulfide nanomaterials. *Adv. Energy Mater.* **4**, 1400739 (2014).

43. Ji L*, et al.* A silicon-based photocathode for water reduction with an epitaxial $SrTiO_3$ protection layer and a nanostructured catalyst. *Nat. Nanotechnol.* **10**, 84-90 (2015).

44. Andoshe DM*, et al.* A wafer-scale antireflective protection layer of solution-processed TiO_2 nanorods for high performance silicon-based water splitting photocathodes. *J. Mater. Chem. A* **4**, 9477-9485 (2016).

45. Zhang H*, et al.* A p-Si/$NiCoSe_x$ core/shell nanopillar array photocathode for enhanced photoelectrochemical hydrogen production. *Energy Environ. Sci.* **9**, 3113-3119 (2016).
