# Supplementary material for: Pt nanoclusters on GaN nanowires for solar-asssisted seawater hydrogen evolution
Source: Nat Commun. 2023 Jan 12;14:179. doi: 10.1038/s41467-023-35782-z (PMC9837051; doi:10.1038/s41467-023-35782-z)
Supplement: Supplementary file 2 — Description to Additional Supplementary Information [file 41467_2023_35782_MOESM2_ESM.pdf]

Description of additional Supplementary Files

Supplementary Movie 1. Photoelectrochemical Seawater hydrogen evolution under concentrated solar light (9 suns) in a liquid flow reactor
